# Alpha-Carbonic Anhydrases from Hydrothermal Vent Sources as Potential Carbon Dioxide Sequestration Agents: *In Silico* Sequence, Structure and Dynamics Analyses

**DOI:** 10.3390/ijms21218066

**Published:** 2020-10-29

**Authors:** Colleen Varaidzo Manyumwa, Reza Zolfaghari Emameh, Özlem Tastan Bishop

**Affiliations:** 1 Research Unit in Bioinformatics (RUBi), Department of Biochemistry and Microbiology, Rhodes University, Makhanda/Grahamstown 6140, South Africa; colleen.manyumwa06@gmail.com; 2 Department of Energy and Environmental Biotechnology, National Institute of Genetic Engineering and Biotechnology (NIGEB), Tehran 14965/161, Iran; zolfaghari@nigeb.ac.ir

**Keywords:** alpha-carbonic anhydrase, homology modelling, motif analysis, MD simulations, dynamic residue network analysis, hydrothermal vents

## Abstract

With the increase in CO_2_ emissions worldwide and its dire effects, there is a need to reduce CO_2_ concentrations in the atmosphere. Alpha-carbonic anhydrases (α-CAs) have been identified as suitable sequestration agents. This study reports the sequence and structural analysis of 15 α-CAs from bacteria, originating from hydrothermal vent systems. Structural analysis of the multimers enabled the identification of hotspot and interface residues. Molecular dynamics simulations of the homo-multimers were performed at 300 K, 363 K, 393 K and 423 K to unearth potentially thermostable α-CAs. Average *betweenness centrality* (*BC*) calculations confirmed the relevance of some hotspot and interface residues. The key residues responsible for dimer thermostability were identified by comparing fluctuating interfaces with stable ones, and were part of conserved motifs. Crucial long-lived hydrogen bond networks were observed around residues with high *BC* values. Dynamic cross correlation fortified the relevance of oligomerization of these proteins, thus the importance of simulating them in their multimeric forms. A consensus of the simulation analyses used in this study suggested high thermostability for the α-CA from *Nitratiruptor tergarcus*. Overall, our novel findings enhance the potential of biotechnology applications through the discovery of alternative thermostable CO_2_ sequestration agents and their potential protein design.

## 1. Introduction

The accumulating concentrations of greenhouse gases (GHGs) over the years have led to the warming of the earth’s atmosphere [1,2,3]. CO_2_ is considered to be one of the GHGs contributing to a significant amount of global warming, with the major source of these gases being the combustion of fossil fuels, rice paddies, and livestock fields [3,4,5,6]. CO_2_ concentrations have increased from approximately 280 ppm (parts per million) in the pre-industrial period to approximately 410 ppm in 2019 [7]. Thus, the discovery and implementation of mitigation strategies is crucial. 

Sequestration of CO_2_ via biomineralization, which involves the aqueous precipitation of minerals in the presence of CO_2_ to form mineral carbonates, is a storage strategy currently being explored [8,9]. The use of a catalyst in this process is important, because otherwise the hydration of CO_2_ is very slow [10]. Some of the reactions that take place during biomineralization, however, require conditions, including high temperatures exceeding 100 °C and an alkaline pH [11,12]. Consequently, thermo-alkali stable enzymatic catalysts are being sought.

Carbonic anhydrases (CAs) are enzymes responsible for the reversible catalytic reaction between CO_2_ and H_2_O. They are viable CO_2_ sequestration agents due to their fast CO_2_ hydration with catalytic turnover rates (*k*_cat_) exceeding 10^4^ s^−1^ for the majority of CAs [11,13,14]. They are metalloenzymes containing a Zn^2+^ metal ion in the active site, with some having shown the ability to utilize iron (Fe^2+^) or cobalt (Co^2+^) instead of Zn^2+^, and still maintain their catalytic activity [15,16,17]. Three major classes of the CA family, α, β, and γ, have been widely studied. The other classes identified include δ, ζ, η, θ, and ι-CAs, with the ι-CAs being the most recently discovered [18,19,20,21,22].

**Figure 1:**
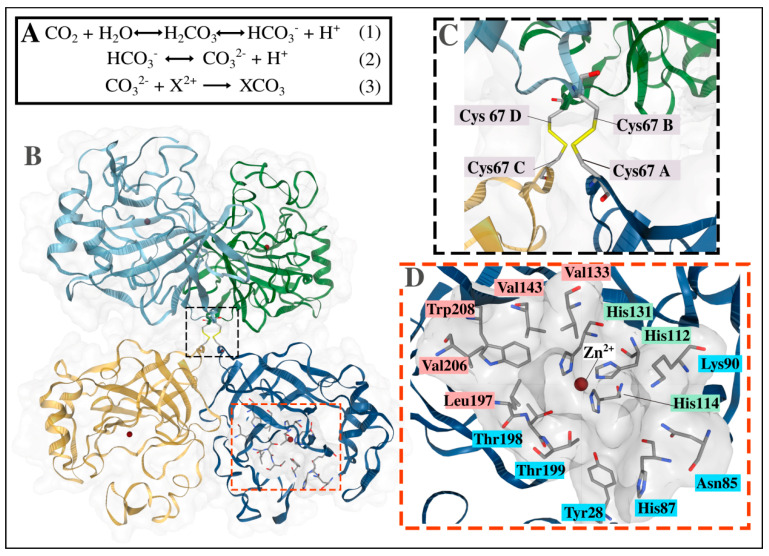
Biological assembly of the alpha-carbonic anhydrase (α-CA) from *Thermovibrio ammonificans* (TaCA, PDB ID: 4C3T). (**A**) illustrates the equations for CO_2_ hydration and dehydration (1 and 2) as well as metal carbonation (3), while (**B**) shows the tetrameric structure of TaCA. (**C**) shows the disulfide core formed by four Cys residues in the tetramerization interface. The catalytic cavity is enlarged in (**D**), with CO_2_ binding pocket residues colored red, proton transfer residues in blue and Zn^2+^ coordinating His residues colored green.

CO_2_ hydration proceeds as shown in Reaction (1) (Figure 1A), resulting in the formation of carbonic acid which dissociates to bicarbonate and hydrogen ions. After being released from the catalytic site into the solvent, the bicarbonate ions further dissociate to carbonate ions (Reaction (2) in Figure 1A) which then react with the metal ions (shown as X^2+^), such as Ca^2+^ or Mg^2+^, during biomineralization to form the mineral carbonate XCO_3_ [10,23]. This is illustrated in Reaction (3) (Figure 1A).

α-CAs are the most widely studied class of carbonic anhydrases, and have been found in mammals, algae, and plants, with numerous reports in bacteria as well [24,25,26]. Most human α-CAs have monomeric structures, in contrast to their bacterial counterparts which have been revealed to have a dimeric assembly [27,28]. A unique tetrameric assembly, absent in all other α-CAs to date, has been observed for the α-CA from *Thermovibrio* *ammonificans* (TaCA, PDB ID: 4C3T) (Figure 1B), which is held together by a core of two disulfide bonds (Figure 1C) [29]. Each monomer has a functional independent active site, containing a Zn^2+^ metal ion in tetrahedral coordination by three His residues as well as a water molecule [14,30,31,32]. They also have a hydrophobic pocket close to the active site where the CO_2_ molecule is normally held during catalysis (Figure 1D). This pocket has been identified in most α-CAs, including the human CA II [33,34,35,36,37].

Given the high environmental temperatures, CAs from hydrothermal vents may already possess attributes to withstand the extreme conditions in the CO_2_ sequestration process, thus providing a viable alternative to engineering CAs. This has been observed in vitro on α-CAs from hydrothermal vent bacteria *Caminibacter mediatlanticus* (CmCA)*, Persephonella marina* (PmCA) and TaCA, with CmCA and PmCA revealing stability up to 70 °C and 100 °C, respectively, and the wild-type and variants of TaCA appearing stable up to 95 °C [38,39,40,41]. Another CA, found in previously isolated DNA from the Logatchev hydrothermal field (LOGACA), has also proven to be thermostable, withstanding temperatures up to 103 °C [35]. The structures of these α-CAs, except CmCA, have been experimentally solved and reported [29,35,42]. Consequently, the *in silico* analysis of the thermostability attributes of α-CAs from hydrothermal vents, is the focus of this study.

To date, *in silico* studies on α-CAs have been for monomeric forms of the proteins. The multimeric occurrence of these enzyme complexes, which is brought about by the interaction of residues between monomers, a region termed the interface or the buried surface area (BSA), is often overlooked [43,44]. The BSA contributes to function as well as stability of the protein complex with a group of residues, termed “hotspot residues”, contributing significantly to protein stability [45,46]. This study considers their biological assemblies (BAs), revealing the relevance of simulating the CAs in their multimeric states. We report here computationally solved dimeric structures and the analysis of 12 α-CAs, including CmCA, from various bacteria coming from hydrothermal vent systems. Analysis was also included for LOGACA, PmCA and TaCA. Sequence alignments and motif analyses were performed to identify conserved and possible functionally important regions in the proteins, followed by phylogenetic tree construction, to view the evolutionary relationships amongst the CAs [47,48]. Analysis of the CA interfaces revealed the presence of hotspot residues, present in conserved motifs, which contribute to the stability of these proteins. Similar results have been shown in Enterovirus capsids [49]. Furthermore, the importance of some interface residues in protein communication was fortified through average *betweenness centrality* (*BC*) analysis of molecular dynamics (MD) simulation trajectories at 300 K [50]. Hydrogen bond networks centered on high communication residues recognized in average *BC* analysis were identified through hydrogen bond analysis. Patterns of thermostability were monitored for the proteins through the radius of gyration (R_g_), root mean square fluctuation (RMSF), and dynamic cross correlation (DCC) analysis of MD simulations at increasing temperatures, 300 K, 363 K, 393 K and 423 K, and were compared to previously characterized CmCA, LOGACA, PmCA and TaCA. Maintenance of active site cavity compactness, low residue fluctuations and high correlated motions at temperatures of 423 K were conspicuous for *Nitratiruptor tergarcus*’ CA (NtCA). Analysis of inter-subunit hydrogen bonds at all four temperatures showed interface disruptions in some proteins at high temperatures, indicative of fluctuations and reduced thermostability. Overall, the computational approach combined data retrieval, sequence alignment, motif analysis, phylogenetic tree calculations, homology modeling of biological assemblies, protein–protein interface analysis coupled with molecular dynamics simulations, dynamic residue analysis and dynamic cross correlation; and thus brought novel aspects to the field of carbonic anhydrases as carbon dioxide sequestration agents. The novelty of our findings enhances the knowledge base of biotechnology applications through the discovery of alternative thermostable sequestration agents as well as their potential protein design.

## 2. Results and Discussion

Sequence and structural analysis of 15 α-CA proteins, of which 14 were from 13 bacteria and one from isolated DNA, was performed (Table 1 and Table S1). The organisms were previously isolated in or around hydrothermal vents, and were mainly Gram-negative [51,52,53,54,55,56,57]. They are classified under four groups (Table 1): Aquificacea, Campylobacteria (formerly known as Epsilonproteobacteria), Deltaproteobacteria (δ-proteobacteria) and Gammaproteobacteria (γ-proteobacteria) [58,59]. Campylobacteria are the most abundant in these locations [60,61,62].

Confirmation of the origin sites of these organisms was crucial to this study, as some genera are not confined to inhabiting hydrothermal environments alone. For example, the genus *Vibrio* is widespread in marine environments [64,65]. Although *Geothermobacter* iron reducers are predominantly confined to hydrothermal systems [66,67,68,69], they have also previously been identified in paddy soils [70]. Bacteria from the families Hydrogenimonaceae, Nautiliaceae, and Nitratiruptoraceae are known to be found entirely in vent systems [71]. 

### 2.1. Sequence Analysis 

The reference sequence from PmCA will be used for α-CA residue numbering going forward unless stated otherwise. All corresponding residues for other sequences are outlined in Table S2.

#### 2.1.1. Multiple Sequence Alignment Reveals the Extent of Conservation in the α-CA Sequences

**Figure 2:**
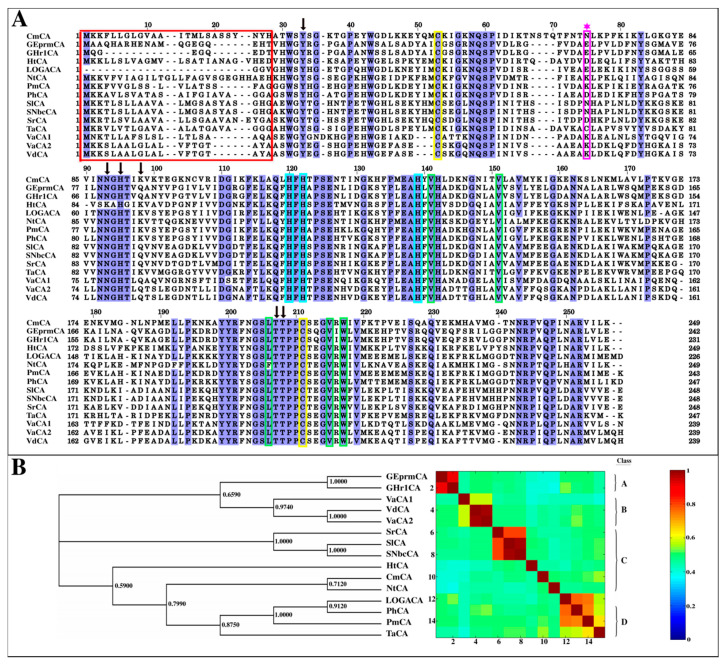
Multiple sequence alignment (MSA) and phylogenetic tree of retrieved α-CA sequences calculated by Tree-based Consistency Objective Function for Alignment Evaluation (T-Coffee) and MEGA7, respectively. (**A**): The red box indicates the signal peptide residues removed during modelling. Proton transfer residues are indicated by the arrows above them and the other functional residues are color coded: cyan—Zn^2+^ coordinating residues; green—CO_2_ binding pocket residues; yellow—Cys–Cys disulfide bond residues. The magenta star indicates the Cys position in TaCA responsible for its tetramerization. (**B**): The evolutionary relationship amongst the 15 retrieved sequences was inferred using the Maximum Likelihood method under the WAG + G + I model and a 100% gap deletion. Bootstrap values from 1000 bootstrap replicates are shown as decimals at their respective nodes. The heat map for the all-versus-all pairwise sequence identity calculations, generated using the T-Coffee MSA, is displayed next to the phylogenetic tree with the magnitude of identity between sequences increasing from 0, shown by the blue color, to 1, shown by red. Classes A, B, C and D indicate the bacteria classes Deltaproteobacteria, Gammaproteobacteria, Campylobacteria and Aquificacea, respectively.

Multiple sequence alignments (MSAs) are important in the identification of conserved regions, which are assumed to be structurally and functionally significant. The MSA produced by Tree-based Consistency Objective Function for Alignment Evaluation (T-Coffee) [72,73] gave an accurate alignment of functional residues across all species and was used for further analysis of the α-CAs. The three His residues that coordinate the Zn^2+^ metal ion in the active site, His107, His109 and His126, were conserved across all sequences and are denoted in Figure 2A by the cyan boxes. CAs of this class have been reported to contain Cys residues (Cys44 and Cys197) that form an intra-subunit disulfide bond affecting structural stability [29,34,74,75,76,77]. These are usually the only two Cys residues in the sequence, indicated by the yellow boxes in the MSA, and were present in all the α-CA sequences sampled (Figure 2A). TaCA, however, possesses a third Cys residue in position 67 (TaCA numbering), which is responsible for its tetrameric quaternary structure [29]. This Cys residue forms a disulfide bond with another Cys in the same residue position of another subunit, resulting in a core of two disulfide bonds by four monomers (Figure 1C) [29]. The rest of the sequences, though of similar origins, and other previously characterized α-CAs, do not possess that particular Cys residue, as shown by the magenta-colored star in Figure 2A. The corresponding amino acid position in the other sequences is moderately variable, with the residue Lys or Asn being the most common substitute. A dimeric architecture, compared to TaCA’s tetrameric structure, was thus assumed for the rest of the bacterial CAs during homology modelling (Section 2.2.1). 

The hydrophobic pocket necessary for CO_2_ binding has been reported in numerous α-CAs, and was present in all structures modelled [27,30,34,78,79]. It contains the hydrophobic residues Val128, Val138, Leu192, Val201, and Trp203 [29,37,75]. All these residues, except Leu192, were completely conserved across the sequences. In NtCA the Leu was substituted by Phe, which is also a hydrophobic amino acid, thus maintaining the hydrophobicity of the pocket. His82 has been reported to be involved in proton shuttling, and was conserved across all the sequences [29,77]. This residue, along with other residues involved in proton transfer as indicated in Figure 2A, as well as Thr83, are part of a cavity including the active site and the hydrophobic CO_2_ binding pocket (Figure 1D). The proton transfer residues form a pocket, referred to as the tertiary CO_2_ binding pocket in hCA-II, which has also been previously observed binding the inhibitor acetazolamide in TaCA [28,29,80,81,82].

#### 2.1.2. Signal Peptides in Most α-CA Sequences Are Confirmed by Signal Peptide Prediction Servers

The cell wall of Gram-negative bacteria is known to be encircled by an outer membrane with a periplasmic space in between [83]. The signal peptide identified in α-CAs is believed to be a useful coping mechanism for the secretion of the CAs, either into the periplasmic space or extracellularly. There it executes CO_2_ hydration, thus, aiding the movement of bicarbonate through the cell membrane [14,29,84]. This is in contrast to the β-CA and γ-CA classes that are found in the cytoplasm [14]. Previously isolated bacterial α-CAs, including LOGACA, PmCA and TaCA, possess signal peptides at the N-terminal of the protein [29,35,85]. In this study, probable cleavage sites for the retrieved sequences were calculated by two signal peptide prediction programs. SignalBLAST [86] uses the BLAST package to predict signal peptides, comparing the query sequence to reference data, whereas Phobius [87] predictions are based on the hidden Markov model (HMM). Results are outlined in Table S3. Predictions from Phobius were in agreement with those from SignalBLAST concerning the absence of a signal peptide in both *Geothermobacter* α-CAs, suggesting cytoplasmic localization for these particular CAs. This observation was also supported by motif analysis (Section 2.1.4) where Motif 11, which was perceived as an indication of a signal peptide, was found to be absent in both *Geothermobacter* spp. CAs. The signal peptide sequence (see Figure 2A), was therefore excluded from the calculation of all structures except the two mentioned above during the modelling of α-CAs in Section 2.2.1. Residues excluded from the modelling of GEprmCA and GHr1CA were as a result of their absence in the template structure, which also possessed a signal peptide. Phylogenetic tree calculations, however, proceeded with complete sequences including the signal peptide.

#### 2.1.3. Evolutionary Relationships amongst the α-CAs Through Construction of a Phylogenetic Tree

The top three models had Bayesian Information Criterion (BIC) values of 7788, 7796 and 7806, respectively. The phylogenetic relationship amongst the retrieved α-CA sequences was inferred by MEGA under the best-fit protein substitution model, WAG + G + I, with a gap deletion of 100%. Phylogenetic analysis evidenced the clustering of the bacteria to match their all-versus-all pairwise sequence identities (Figure 2B). The pairwise identity heat map, which was derived from the MSA in Figure 2A, revealed high sequence identities in CAs belonging to similar lineages. CAs which belonged to the *Sulfurovum* genus had high sequence identities (above 80%) and clustered together. VaCA2 and VdCA were highly similar, in contrast to the comparison of either of the sequences to VaCA1. The *Geothermobacter* spp. CAs also showed high sequence identities (above 80%). Tree branching suggested that unclassified LOGACA belonged to the class Aquificacea and genus *Persephonella.* This α-CA showed the closest relation to PhCA, with a sequence identity of 87% and a branch bootstrap value of 0.912. Clustering patterns by some of the sequences align with phylogenetic tree calculation results obtained by Nakagawa et al. [57]. Common bacteria to both studies include *H. thermophila, N. tergarcus*, *S. lithotrophicum*, and *Sulfurovum* sp. NBC 37-1. Trees were calculated using 16s rRNA sequences and branching of these bacteria proved similar to those observed in this study [57].

#### 2.1.4. Motif Analysis Reveals Functionally Important Motifs as Well as Conservation across Sequences

**Figure 3:**
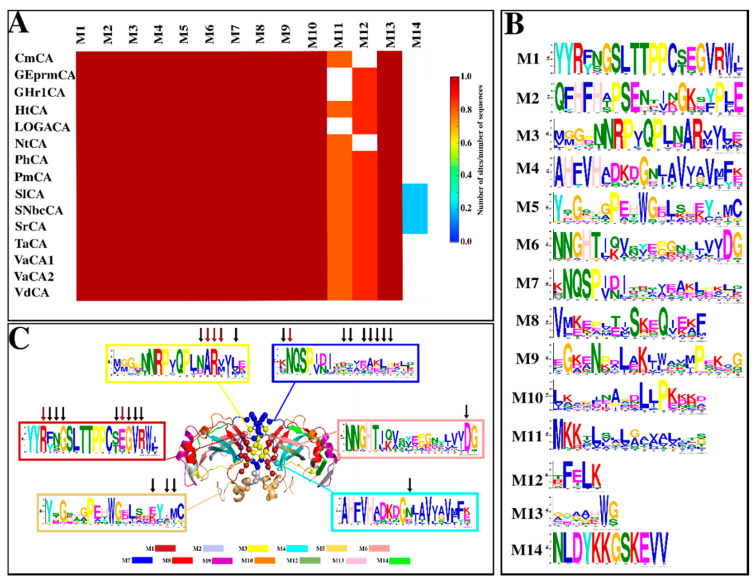
Motif analysis of the α-CA sequences sampled. (**A**): Heat map showing the frequency of each motif and coloring is based on the extent of conservation of the motif across the sequences. (**B**): Motif numbering and the motif web logos, which depict the extent of the amino acid conservation in each position, are based on results generated by Multiple Expectation Maximisation for Motif Elicitation software (MEME). Residue colors are indicative of their chemical properties as follows: blue–most hydrophobic residues (A, C, F, I, L, M, V and W); red–positively charged residues (K and R); green–polar, non-charged and non-aliphatic residues (N, Q, S and T); magenta–most acidic residues (D and E); light pink, orange, turquoise and yellow are for H, G, Y and P respectively. (**C**): Motifs are mapped onto the structure of *Sulfurovum lithotrophicum* (SlCA). Identified interface residues are shown as spheres and colored according to the color of the motif on which they are found. Motif web logos displayed in (**C**) are those for motifs containing interface residues. Motif 11 was omitted due to its absence in the modelled structures. Interface and hotspot residues are indicated by the black and red arrows, respectively.

Motif analysis was performed to elucidate conserved patterns amongst the sequences. 14 unique motifs were identified for the α-CAs and generally, a high conservation of motifs across the sequences was perceived, with 11 being conserved in the dataset (Figure 3). Several motifs have been identified as functionally important, as they contain residues that are critical to the function of the CAs (Table 2). Motif numbering is aligned to results produced by Multiple Expectation Maximisation for Motif Elicitation (MEME) software [88]. Start and end positions of motifs for each sequence, as well as motif E-values, are outlined in Table S4. 

Functional residues previously identified were observed and highly conserved in the MSA of the α-CAs in this study and were located in various motifs (Table 2). Residues in the interface of the dimers (Section 2.2.2) also signified the structural importance of some motifs and are included in Table 2. Asn80 and Lys85 are proton shuttling residues present in Motif 6, with HtCA possessing a Lys residue in place of Asn80. Lys85 position was occupied by either a Lys or Gln residue in the retrieved sequences. The α-CAs that possessed Gln in this position included both *Geothermobacter* CAs, all three *Sulfurovum* α-CAs and all three *Vibrio* α-CAs. The rest had Lys in that position. Functional importance of motifs 8, 9, 10, 12, 13 and 14 is unknown. Motif 11 was located at the N-terminal, except in the *Geothermobacter* spp. α-CAs, correlating with signal peptide predictions in Section 2.1.2, as this motif was observed to be part of a signal peptide with residues 1 to 16.

### 2.2. Homology Modelling and Structural Analysis

3D protein structures were calculated via homology modelling. These structures were then used for mapping sequence and motif information, structural analysis, and MD simulations which helped to understand the differences between monomeric and multimeric forms of the proteins, as well as the identification of key interface residues. 

#### 2.2.1. Calculations of Dimeric Models of α-CAs and Their Validation Yields Good Quality Structures

The crystal structure of LOGACA was used as the template for the dimeric structure calculations of all sequences retrieved except PmCA and TaCA, which already have crystal structures. LOGACA had a resolution of 2.5 Å and good coverage with all the CA sequences, which ranged between 87% and 95%. Sequence identities with the retrieved sequences varied between 47% and 84% (Table S5). The signal peptide sequence, also absent in LOGACA, was excluded from the models, along with a few residues that were not structurally available from the template and these are indicated by the red box in Figure 2A. The starting residue numbers for each of the structures after alignment trimming during modelling are noted in Table S3 along with suggested cleavage sites from the signal peptide prediction servers. Structures were modelled with an active site Zn^2+^ metal ion tetrahedrally coordinated by three His residues and a water molecule. Coordinating His residues were all within the expected bond length of 2.2 Å to Zn^2+^ [89]. The active site appeared to be open and accessible to the solvent as observed in most CAs, with the catalytic pocket extending from the outside of the protein to the metal ion [34,90,91]. Validation results from various programs revealed that the structures modelled were of good quality (Table S5). The z-DOPE (Discrete Optimized Protein Energy) scores were all below the quality threshold of −0.5, with structure quality improving as the score became more negative. The Ramachandran plots generated using PROCHECK [92] showed that 87% or more of residues for each of the structures were in the most favored regions. Verify3D [93] compares the protein sequence (1D) to the 3D structure, producing results as a percentage of residues with scores above 0.2. Percentages above 80% are considered a pass, and all α-CA structures passed this verification.

#### 2.2.2. Structural Analysis of the α-CA Multimers Reveals Hotspot Residues in the Protein Interface and Important Inter- and Intra-Subunit Interactions

It was crucial to investigate the interface residues of the multimeric α-CAs and their interactions, particularly hotspot residues, mutation of which might result in destabilization of the protein [45,94]. The residues involved in interface formation, as well as hotspot residues as a subset of the interface residues, were identified using various web servers (Table S6). In TaCA, two different interfaces were considered; the dimerization interface, which is between two monomers forming the bacterial dimer, and the tetramerization interface, which is between the dimers responsible for tetramer formation. Henceforth, residues from a neighboring monomer will be signified by an asterisk (*). 

It was interesting to observe marginal asymmetry in the spatial orientation of residues in the interface. On account of this, similar residues in different chains were found to be contributing differently to the interface binding energy, thus one could be identified as a hotpot residue but not the other. Complete conservation was observed in the sequence alignment (Figure 2A) for Asn49, Arg187, Ala237 and Arg238, which appeared as interface residues across all sequences. The important inter-subunit interactions formed by these residues were deduced from the Protein Interactions Calculator (PIC) web server [95]. Asn49 was observed to form hydrogen bonds with a number of residues including Asn49 * and Ser189 * as well as Glu199 *. Variability in Ser189 position did not affect the presence of the Asn49–Ser189 * bond in the CAs. Glu199 also formed hydrogen bonds with the same residue in the neighboring monomer. In *Geothermobacter* spp., Glu199 was replaced by a Gln residue, but showed similar interactions in the interface. The Arg187–Ala237 * hydrogen bond in the interface was common to all proteins. The combination of Arg187–Asp102 and Asp102–Arg207 intra-subunit salt bridges (TaCA numbering) has been previously recognized in TaCA and is referred to as an ion-pair ‘latch’ [29]. This was observed for the α-CAs in this study, except the *Geothermobacter* spp. α-CAs, which lacked an Asp102–Arg207 salt bridge due to an Ile substitution in place of Arg207. 

*Sulfurovum lithotrophicum*’s α-CA (SlCA) contained all 14 motifs identified; thus, it was used as a representative structure in Figure 3C for the mapping of motifs and interface residues, which are outlined in Table S6. This was in order to visualize the interface, the motif positions, and their interactions. High variability of most interface residues in Motifs 5 and 7, besides hotspot residue Asn49, was observed. Physicochemical properties in each position varied across the sequences with possible implications to individual stabilities of the proteins, which were searched further through MD simulation analyses (see Section 2.4.3). Characteristics of the interfaces obtained from the Protein Interfaces, Surfaces and Assemblies (PDBePISA) web server [96], including the number of hydrogen bonds, salt bridges formed as well as the BSA of each model, are listed in Table S7. Inter-subunit hydrogen bonds and salt bridges were also queried from the PIC web server [95]. Results from PDBePISA revealed the protein subunits to be held together by approximately 7–18 inter-subunit hydrogen bonds of moderate strength with H-bond distances varying between 2.6 Å and 3.7 Å. Salt bridges are bonds between groups of opposite charges between the negative carboxyl oxygen atom from either Asp or Glu residues and the positive nitrogen atom from either His, Lys or Arg residues within 4 Å of each other [97,98]. Their presence in protein structures has been recognized as potentially stabilizing, and possibly contributing to and increasing their thermostability [99,100]. Both PIC and PBDePISA concurred that CmCA*,* both *Geothermobacter* CAs, HtCA, VaCA2 and VdCA did not contain any inter-subunit salt bridges.

The BSA and the total solvent accessible area of each dimer was calculated in Å^2^ and both are shown in Table S7. CmCA*,* HtCA and PmCA had the largest buried surface areas of 2094.6 Å^2^, 2082.8 Å^2^ and 2011 Å^2^, respectively. Dimeric buried interface residue areas are similar to those reported for the thermophilic α-CA species *Sulfurihydrogenibium azorense* (PDB ID: 4 × 5S) (2050 Å^2^), with that of *Sulfurihydrogenibium yellostonense* (PDB ID: 4G7A) being much higher at 2300 Å^2^ [34,74]. In comparison to the retrieved sequences, the α-CA from the mesophile *Neisseria gonorrhoea* (PDB ID: 1KOP) was also put through PISA, and was observed to have a smaller buried surface area of 1691 Å^2^.

### 2.3. Dynamic Characterization of the Proteins

**Figure 4:**
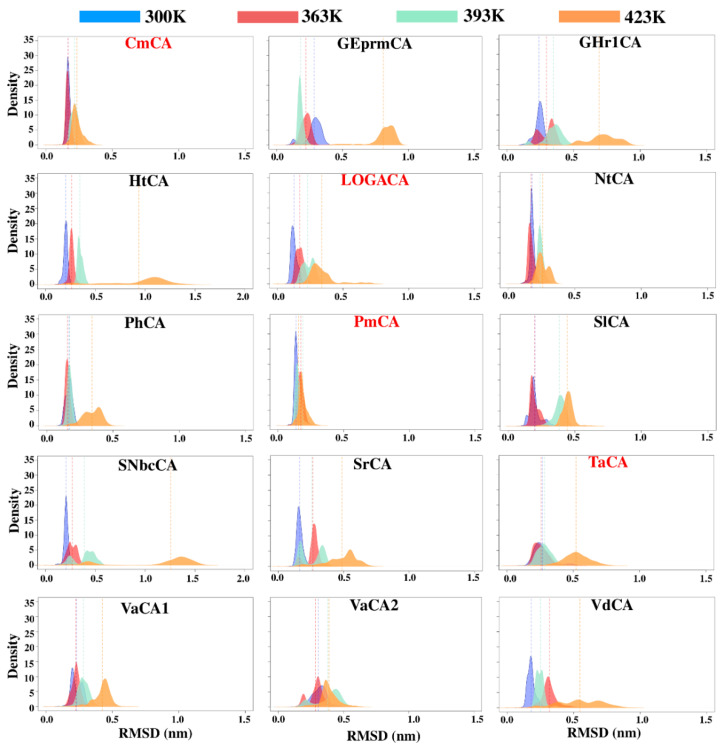
Kernel density estimation plots for root mean square deviation (RMSD) of the α-CA proteins simulated at 300 K, 363 K, 393 K and 423 K. The average RMSD of each histogram is shown as a dotted line colored the same as its respective histogram. The CA from the Logatchev hydrothermal field, LOGACA, as well as CmCA, PmCA and TaCA which have been previously characterized, are labelled in red.

Although the analysis of static multimeric models gave an adequate representation of the important interactions within the structures, proteins often have a number of conformations in vivo, thus necessitating their simulation and analyses [101]. MD simulations allowed sampling of a wide range of these conformations, as shown by the root mean square deviation (RMSD) Kernel density estimation (KDE) plots in Figure 4. All simulations had equilibrated by 40 ns (Figure S1), substantiating the 50 ns runs. Average *BC* and hydrogen bond analysis of the simulations at room temperature (300 K) gave a clearer picture of the residues with important roles during these conformational fluctuations. 

#### 2.3.1. Dynamic Residue Network Analysis Confirms Interfacing and Hotspot Residues along with Previously Identified Functional Residues

Dynamic residue network (DRN) analysis is an approach of MD-TASK designed to observe the changes in the communication of protein residues during an MD simulation [50]. The C_β_ atoms (C_α_ for Gly) of residues are represented as nodes as part of this network and the shortest path to each node is calculated. Average *BC* calculations were performed to identify residues important in the communication of these α-CAs. Distributions of the average *BC* values across the proteins were positively skewed, thus relevance of high communication residues was considered for the top 5% residues showing the highest average *BCs* and these are listed in Table 3. Some of the hotspot and interface residues that we identified (Section 2.2.2) were observed to have significant average *BC*s, displaying frequent usage and noteworthy importance in the functioning of the α-CAs. It was observed that the asymmetric behavior displayed during interface analysis of static structures was more pronounced in the MD simulations. Similar residues in different chains showed different average *BC* intensities (Figure S2), thus in all proteins, residues appearing above the 5% cut-off were marginally different for monomers of the same protein. Hotspot residue Asn49 showed relatively high average *BC* compared to other residues in their respective structures in the average *BC* analysis of most proteins. Interface residue Asn194 (SlCA numbering), which was not found in α-CAs from Aquifaceae and NtCA, was also found above the cut-off. Residues with high average *BC*s in TaCA were observed to be located more towards the tetramerization interface, with the Cys residues that formed the two inter-subunit disulfide bonds displaying the highest usage. These disulfide bonds have been shown to be crucial for the thermostability of TaCA [29]. Val64 and Leu68 (TaCA numbering) had relatively high *BC*s and were observed to form hydrophobic interactions in the dimerization interfaces of both dimers. Contributing to the tetramerization interface was Lys65, forming hydrogen bonds with Lys247 in the opposite monomer in a different dimer.

Amongst the residues identified were also those previously identified in literature as crucial to the catalytic activity of the α-CAs. CO_2_ binding pocket residue Trp211 (CmCA numbering) showed a high usage in most proteins. Of particular interest was Val201, which was observed to play a role in the dimer interface as well as operating as a CO_2_ binding pocket residue. This occurrence was observed across all the proteins simulated with relatively high average *BC*. Functionality of the residues without annotation in Table 3 was unknown from previous literature and was further searched using hydrogen bond analysis.

#### 2.3.2. Intra-Subunit Hydrogen Bond Analysis Reveals Important Networks Going through High Communication Residues Identified in Average *BC* Analysis

Hydrogen bonds play a key role in protein stability [102,103]. Analysis of intra-subunit bonds substantiated the presence of high communication residues identified in average *BC* analysis, whose function was not recognized from previous literature. These residues were queried from the results produced by *cpptraj* [104] and the networks shown in this study were constructed on the basis of persistent hydrogen bonds, i.e., bonds lasting more than 90% of the simulation, around the residues. All proteins were observed to have a hydrogen bond network around Glu124, which appeared in the top 5% for most CAs in average *BC* analysis. This network involved at least two high communication residues in the proteins, except in HtCA where the Glu residue was the only one. For all proteins, it included hydrogen bonds between Glu124 and residues His109 and His126, which are functional as Zn^2+^ coordinating residues. GEprmCA and GHr1CA had the greatest number of high communication residues, i.e., four, in a single hydrogen bond network (Glu124, Ser140, Asn114 and Val53—GEprmCA numbering). 

**Figure 5:**
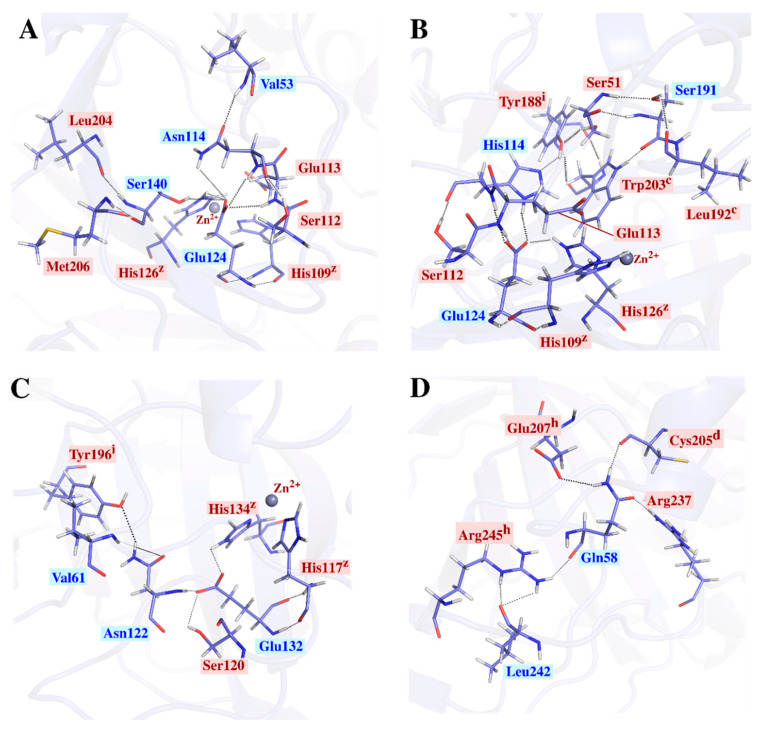
Hydrogen bond networks around some residues with high average *BC* (labelled in blue) confirmed through average *BC* analysis. Networks shown for GEprmCA (**A**), PmCA (**B**) and NtCA (**C**), respectively, pass through the conserved Glu132 (NtCA numbering). (**D**) shows the hydrogen bond network around NtCA’s Gln58. All bonds were present for > 90% of the simulation. Functional residues are annotated as follows: hotspot residues—h; interface residues—i; intra-subunit disulfide Cys residues—d; CO_2_ binding pocket residues—c; Zn^2+^ coordinating residues—z.

An illustration of GEprmCA’s Glu network is shown in Figure 5A. The largest hydrogen bond network, with 11 residues interacting for most of the simulation, was observed for thermostable PmCA (Figure 5B) and LOGACA (Figure S3K) as well as for PhCA (Figure S4D), which all belong to the same class. Amongst these 11 residues were CO_2_ binding pockets Trp203 and Leu192, both of which formed hydrogen bonds with high communication residue Ser191. The Leu192–Ser191–Trp203 network was also present, but observed to be separate from the Glu124 network in CmCA, HtCA, SlCA and SrCA (Figures S3D,J and S4H,L). Apart from its Glu network shown in Figure 5C, NtCA was observed to possess a second unique hydrogen bond network (Figure 5D), centered on Gln58 and including Leu242 (NtCA numbering), both which showed high usage in average *BC* analysis. Persistent hydrogen bonds were observed between Gln58 and hotspot residues Glu207 and Arg245, as well as intra-subunit disulfide bonds Cys205 and Arg237 (NtCA numbering). Interactions formed by Glu207 (NtCA numbering) in the interface were probed and mentioned in the interface analysis (Section 2.2.2) This additional network is presumed to be of weighty importance towards stability of the protein both within and across subunits. Hydrogen bond networks for the rest of the structures are shown in the (Supplementary Data Figures S3–S5).

### 2.4. Identification of Potentially Thermostable α-CAs Using High Temperature Simulations 

Simulation of the proteins at increasing temperatures of 300 K, 363 K, 393 K and 423 K in this study unearthed other potentially thermostable α-CAs. Behavior patterns of the uncharacterized CAs were compared to those of CAs already pre-determined to be thermostable, i.e., CmCA, LOGACA, PmCA and TaCA, upon trajectory analyses. Similar patterns, and possibly those displaying higher thermostability properties, were seen for some of the proteins in these analyses, which included R_g_, RMSF, DCC and inter-subunit hydrogen bond analysis. Results from the analysis of the temperature MD simulations also further substantiated the importance of multimeric simulations.

#### 2.4.1. Compactness of the Chains and Catalytic Cavities from Each Protein Is Assessed by R_g_ Analysis at Increasing Temperatures

**Figure 6:**
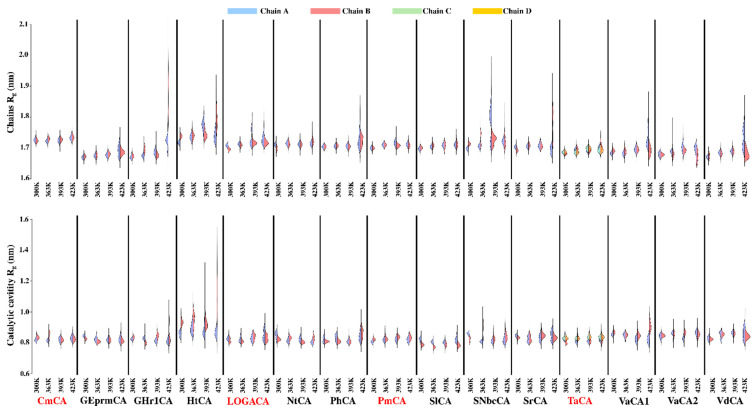
Radius of gyration (R_g_) of the α-CAs’ individual chains and catalytic pockets at 300 K, 363 K, 393 K and 423 K. CmCA, LOGACA, PmCA and TaCA, which have been previously characterized, are labelled in red.

The cavity with Zn^2+^ contains all the catalytically important residues, including those forming the CO_2_ binding pocket, His coordinating residues, and proton transfer residues. Maintenance of this cavity at high temperatures is thus perceived as important for the sustenance of catalytic activity in these extreme conditions. R_g_ analysis, which assesses protein compactness, was used to monitor the compactness of (i) the separate chains and (ii) the catalytic cavity of each chain across all temperature trajectories; these are illustrated in Figure 6. The length of the vertical lines represent the minimum and maximum R_g_ exhibited at each temperature. This approach was recently used for the analysis of the active and allosteric sites of the main protease of SARS-CoV-2 [105]. Although the CAs are homo-multimers, the behavior of either the individual chains, the pockets, or both, was asymmetrical at one or more temperatures for all proteins. This was, however, less evident for CmCA, SlCA and TaCA. At all four temperature simulations, compactness of the catalytic cavities was maintained for CmCA, GEprmCA, NtCA, PmCA, SlCA and TaCA, an indication of resisting denaturation at high temperatures, and possibly the maintenance of catalytic activity. Decrease in compactness was noted for GHr1CA and HtCA’s catalytic cavities as well as their chains with an increase in temperatures. Despite a general increase in R_g_ for SNbcCA’s chains A and B at 393 K, it was interesting to observe a maintenance of compactness for the catalytic pockets. This was also seen for GHr1CA’s and SrCA’s chain B at 423 K, as well as for VaCA2.

#### 2.4.2. RMSF Analysis Reveals Stability of Some Proteins at High Simulation Temperatures

RMSF is a measure of average residue displacement across a trajectory compared to its position in the initial frame. The effects of increasing temperature on individual residue fluctuations in the multimeric structures were investigated using RMSF analysis. Rigidity of the residues at lower temperatures is a desirable characteristic for increased thermostability and is necessary to make up for fluctuations at high temperatures found in the CO_2_ sequestration process [106,107]. The N-terminal is known to be a generally flexible region and exhibited high residue fluctuations in most proteins, particularly at 393 K and 423 K (Figure S6). This flexibility could also be accredited to the removal of the signal peptide identified in Section 2.1.2. Reference α-CAs CmCA and PmCA displayed low fluctuations at all four temperatures. These proteins are known to possess high thermal stability properties; thus, a similar effect was explored in the proteins simulated as a speculation of thermostability, and was observed for NtCA. At 423 K, marginally higher fluctuations were observed for LOGACA compared to lower temperatures, and a similar behavior was noted for GEprmCA, PhCA, SlCA, SrCA and VaCA1. The highest fluctuations were seen at 423 K for VdCA, HtCA, GHr1CA, HtCA and SNbcCA, in order of increasing fluctuations. These results implied a reduced tolerance to high temperatures due to losses in structural rigidity compared to the other α-CAs.

#### 2.4.3. Disruption of the Interfaces in High Fluctuating α-CAs Is Investigated

**Figure 7:**
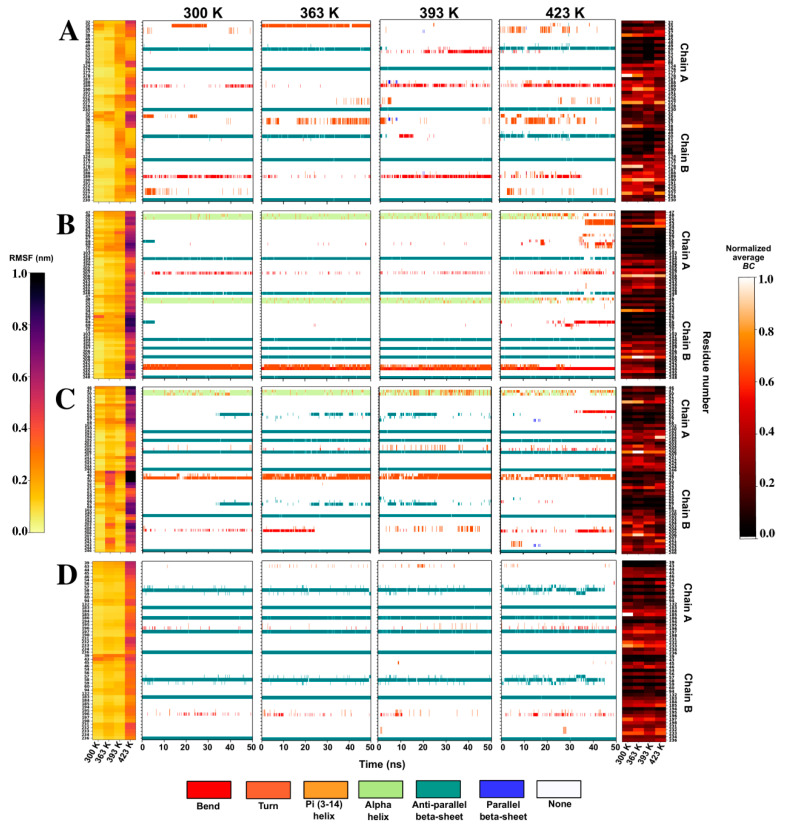
Root mean square fluctuation (RMSF), Define Secondary Structure of Proteins (DSSP) and average *BC* analyses of interface residues of (**A**): GHr1CA (**B**): HtCA (**C**): SNbcCA and (**D**): VdCA at 300 K, 363 K, 393 K and 423 K.

The secondary structures adopted by proteins during folding are important for their function and structural integrity; and consequently, protein unfolding when exposed to extreme conditions is often accompanied by secondary structure loss [108,109,110]. As such, the high fluctuations observed for GHr1CA, HtCA, SNbcCA, as well as VdCA, prompted the investigation of the occurrence of secondary structure elements of each residue over the 50 ns trajectories using Define Secondary Structure of Proteins (DSSP) analysis [111]. Particular attention was paid to the interface residues identified in Section 2.2.2, and this is shown in Figure 7, along with the changes in RMSF as well as the average *BC* of each residue with increases in temperature. RMSF of interface residues for the rest of the structures is illustrated in Figure S7. Loops in protein structures are generally known as regions of high flexibility. Most interface residues in the proteins in Figure 7 were observed to be present in loop regions upon DSSP analysis. Hydrophobic interactions formed between HtCA’s Met48, found in Motif 5, and Ile51, which was not located on any motif, are suggested to prevent solvent accessibility from the N-terminal end of the interface. This is signified by the relatively lower fluctuations observed in the N-terminal region of HtCA at 423 K compared to its other interface residues at this temperature. These hydrophobic interactions were also conserved for CmCA, LOGACA, PhCA and PmCA. The last three CAs however, also contained hydrophobic residue Ile49 (PhCA numbering) thus lower fluctuations were observed at 423 K (Figure S7) compared to HtCA which had hydrophilic Glu instead. Inevitably, disruption of the predominantly alpha helical structure involving Met48 and Ile51 (HtCA numbering), in both HtCA chains was observed at 423 K. Interface residues of HtCA in Motif 7 (Tyr66, Asp67 and Asp69) located on the opposite periphery to Motif 5 interface residues, were amongst the highest fluctuating residues in both chains. These residues create a polar environment in this region, allowing easy solvent accessibility. The remarkable stability of CmCA’s interface towards Motif 7 was observed to be brought about by the hydrogen bonds formed by Asn68–Thr69, Thr64–Asp105 and Asn68–Asn70. It is also worth noting that the Cys residues involved in the tetramerization disulfide bridge in TaCA, which are located in Motif 7, barricade solvent accessibility to the dimerization interface, thus the exceptional interface stability was observed (Figure S7). In VdCA, the degradation of the anti-parallel beta-sheet involving Ala58 was also seen in both chains at 423 K. Differences in the structural propensities of interface residues was also noted between chains, particularly for HtCA (Figure 7B), further highlighting the asymmetric chain behaviors.

Except for the hotspot residue Asn49, the interface residues in Motifs 5 and 7 were observed to be low communication residues. These residues are oriented closer to the N-terminal as well as being obscurely accessible for the solvent (see Figure 3C), compared to those located closer to the C-terminal across all proteins. Asn49, which showed a higher average *BC* compared to these interface residues, was completely conserved across all sequences, whereas the rest in Motifs 5 and 7 displayed very low conservation. Val201 (HtCA numbering) was observed to have higher average *BC* compared to most of the interface residues at most temperatures. Hydrogen bond interactions in the interface are further analyzed in Section 2.4.4.

#### 2.4.4. Effects of Temperature on Hydrogen Bond Interactions in the Interface Are Monitored by Inter-Subunit Hydrogen Bond Analysis

**Figure 8:**
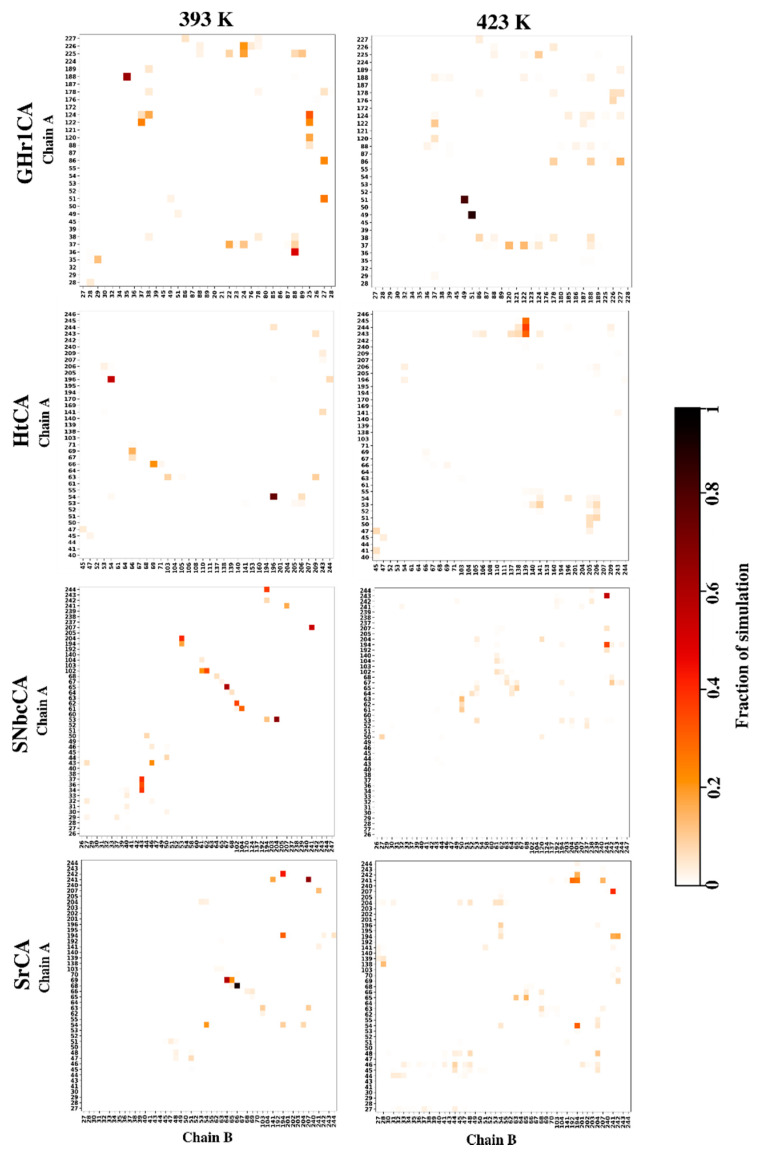
Inter-subunit hydrogen bond analysis of GHr1CA, HtCA, SNbcCA and SrCA at 393 K and 423 K.

Following the identification of interface residues in Section 2.2.2, it was interesting to identify the occurrence of hydrogen bonds in the interface across trajectories with an increase in temperature (Figures S8–S11). During this analysis, it was important to differentiate between the hydrogen bonds contributing to interface stability from those formed by temporary proximity of residues due to fluctuations. The most important hydrogen bonds were identified as those maintained for a larger fraction of the trajectory, whereas numerous short-lived bonds were an indication of residue fluctuations in the interface. The latter was seen for proteins HtCA, SNbcCA and SrCA at 423 K, with an absence of persistent hydrogen bonds being observed at this temperature (Figure 8). HtCA and SNbcCA interface residues were correspondingly showing large fluctuations at 423 K in Section 2.4.3. NtCA showed a number of hydrogen bonds present for most of the simulation at all four temperatures (Figure S9), which led to the low fluctuation in the interface residues (Figure S7). Hydrogen bonds mentioned for CmCA in Section 2.4.3 were observed at all four temperatures (Figure S8), thus, they are confirmed to contribute to interface stability. Upon a closer look at the results, it was observed that Val201, which was identified in the interface in Section 2.2.2 and had a high average *BC,* was not seen forming any hydrogen bonds in the interfaces of all the proteins simulated. Its high average *BC* values across all proteins are therefore accredited to its role in the CO_2_ binding pocket. An increase in short-lived hydrogen bonds was accompanied by the loss of the two Asp60-Lys62 * long-lived hydrogen bonds in PmCA at 393 K and 423 K (Figure S9). In TaCA, the tetramerization interface had adjacent monomers (chain A–D and chain B–C) showing short-lived hydrogen bonds between Lys65-Ala66 * and Lys65-Lys247 * only, with the latter having been identified in average *BC* analysis. The dimerization interfaces contained a consistent hydrogen bond pattern throughout the temperatures, with the most long-lived bonds forming between Lys65 and disulfide bonding Cys67 * (Figure S11).

#### 2.4.5. Correlated Motions Confirm Synchronized Movement of the Dimers through Interfacing Interactions

**Figure 9:**
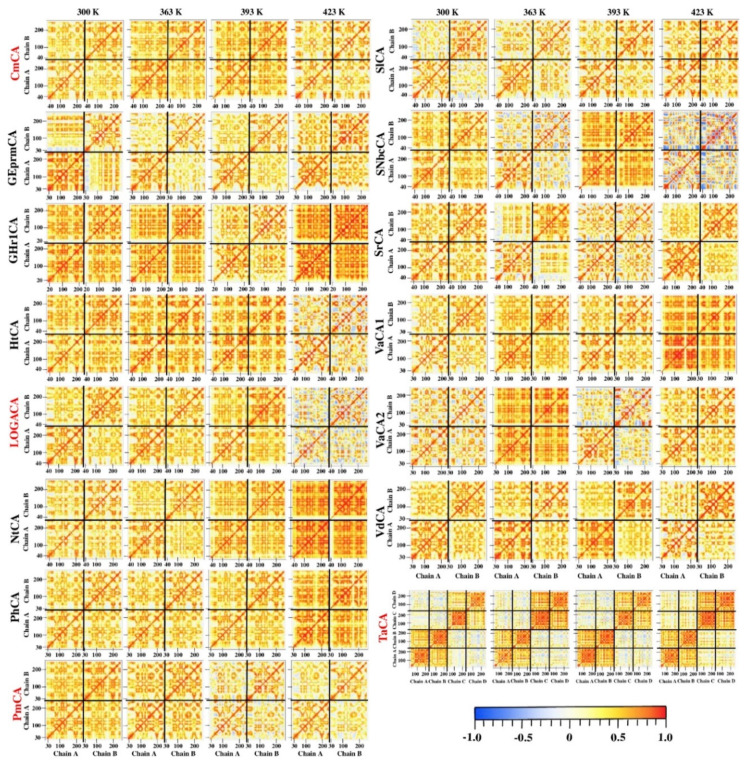
Dynamic cross correlation (DCC) analysis of α-CAs at 300 K, 363 K, 393 K and 423 K. CmCA, LOGACA, PmCA and TaCA, which have been previously characterized, are labelled in red.

To gain insight on the effect of temperature on the synchronized movements between the residues of each protein, the correlation coefficients of motions between the C_α_ atoms were calculated as described in Materials and Methods (Section 3.9). Correlation matrices were plotted as heatmaps to visualize these motion dynamics (Figure 9). Common to all proteins, highly synchronized motions were observed between chains, confirming communication through the interface. This observation also signified the importance of oligomerization to the thermostability of these enzymes, and more so, their simulation in these states. The highest correlated intra- and inter-subunit motions at 423 K were observed for GHr1CA, NtCA, PhCA and VaCA1. Interestingly, GHr1CA was amongst the CAs showing increased fluctuations in RMSF analysis at this temperature. Unsynchronized motions between chains observed at 423 K for HtCA, LOGACA and SNbcCA, as well as the reduced correlations between the chains in PmCA at 393 K and 423 K, were consistent with the increase in intermittent hydrogen bonds observed in Section 2.4.3.

TaCA displayed a behavior slightly different from the dimeric structures. In this tetrameric structure, chains A and B, which are diagonally oriented to each other, were observed to exhibit higher synchronized motions compared to those between chains A and C, which form the bacterial dimer. The same was observed for chains C and D in comparison to the dimer formed by chains B and D. This observation corroborates with the average *BC* analysis results, where the highest communication was observed passing through the Cys residues forming the inter-subunit disulfide bonds. Low anti-correlated motions were present between the adjacent monomers, i.e., chain A–D and chain B–C, forming the dimers in TaCA possibly due to reduced interactions between the chains.

## 3. Materials and Methods 

**Figure 10:**
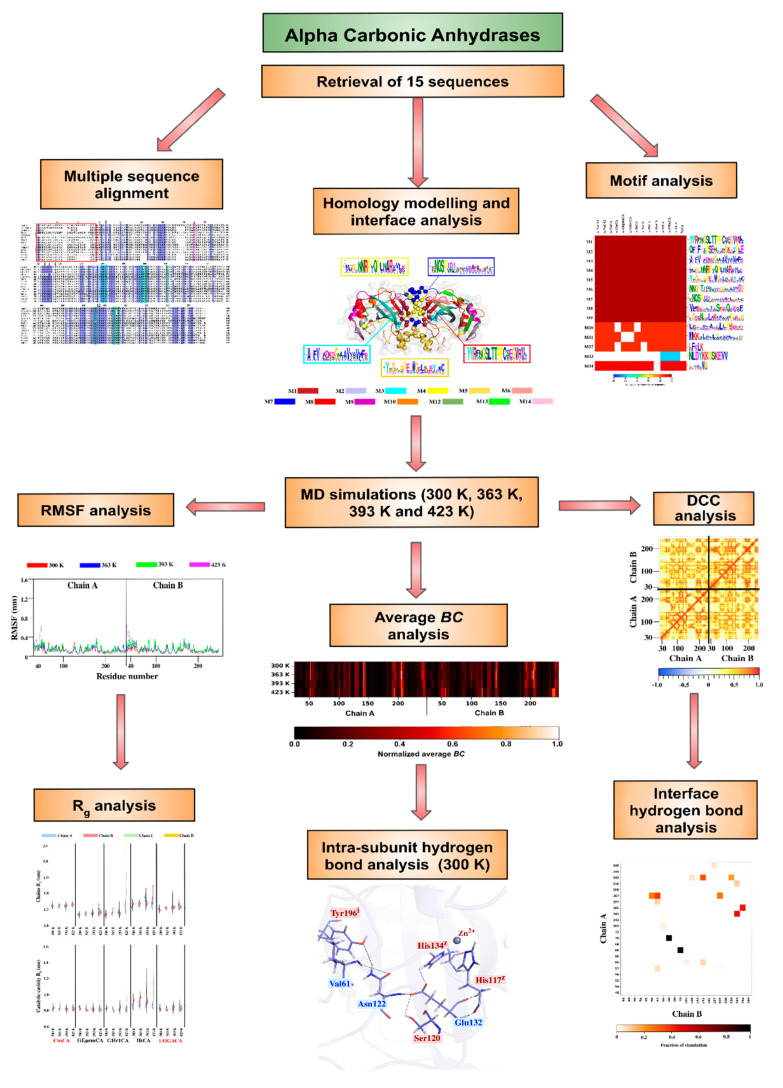
Overall methodology followed in this study. *BC*, DCC, R_g_ and RMSF refer to *betweenness centrality*, dynamic cross correlation, radius of gyration and root mean square fluctuation, respectively.

The overall methodology is shown in Figure 10. 

### 3.1. Sequence Retrieval 

Retrieval of sequences for this study was conducted in two consecutive parts. In the first part, Position-Specific Iterated BLAST (PSI-BLAST) in the National Center for Biotechnology Information (NCBI) [112] was utilized for two iterations to identify sequences that were homologous to the query sequences from three organisms originating from hydrothermal vents. These were CmCA (NCBI Accession Number: WP_007474387), and previously crystallized PmCA and TaCA (NCBI Accession numbers: WP_015898908.1 and WP_013538320.1, respectively) [29,42,51]. The second part of retrieval involved searching for published literature on the origins of the organisms, and only those verified to be from hydrothermal vents were considered. Further sequences that were more than 40% similar to the query, with query coverage above 90% were selected. 

Overall, 15 sequences were identified from the organisms that were previously isolated from hydrothermal vents (Table S1). These organisms included *C. mediatlanticus* [51], *Geothermobacter* sp. EPR-M [67], *Geothermobacter* sp. HR-1 [68], *Hydrogenimonas thermophila* [113], *Nitratiruptor tergarcus* [114], *Persephonella hydrogeniphila* [53], *P. marina* [40,42], *Sulfurovum lithotrophicum* [55], *Sulfurovum riftiae* [56], *Sulfurovum* sp. NBC37-1 [57,115], *T. ammonificans* [29], *Vibrio antiquarius* [116] and *Vibrio diabolicus* [54]. Two different α-CA sequences from the bacterium *V. antiquarius* were included in the list of sequences, as well as the unclassified sequence of α-CA structure LOGACA. 3D structures of LOGACA, PmCA and TaCA (PDB IDs: 6EKI, 6IM3 and 4C3T, respectively) were downloaded from the Protein Data Bank (PDB) [35,52,117,118]. Table S1 summarizes the accession numbers and query coverages, as well as E-values corresponding to α-CAs of each bacterium with the class containing a total of 15 sequences. Sequence lengths and percentage identities to the query sequences are also shown in the table.

### 3.2. Sequence Alignments and Signal Peptide Prediction

Multiple sequence alignment (MSA) was performed using the Tree-based Consistency Objective Function for Alignment Evaluation (T-Coffee) [72,73] and Multiple Alignment using Fast Fourier Transform (MAFFT) [119] alignment programs with default settings, and visualized in Jalview v2 [120]. An all-versus-all sequence identity matrix was produced for the best-fitting alignment by use of an in-house Python script, which tallied the number of identical amino acid positions between each sequence and all other sequences in the dataset, representing the total as a fraction of the sequence length in the matrix [121]. A heat map of the matrix was subsequently generated using GNU Octave v4.0.0 [122].

All α-CA sequences in the dataset, except those for crystallized LOGACA, PmCA and TaCA, were simultaneously submitted for signal peptide prediction using two different prediction servers, Signal-Blast and Phobius [86,87]. The consensus of the servers was used to discern the presence or the absence of a signal peptide in each of the proteins.

### 3.3. Phylogenetic Tree Analysis

MEGA v7 [123] was employed for phylogenetic tree calculations using the T-Coffee MSA as the input. The tree models were generated with gap deletions of 90%, 95% and 100%, using the strong branch filter option. Bayesian Information Criterion (BIC) scores were used to rank the models, and the first 3 models displaying the lowest BIC scores were selected for phylogenetic tree generation. The top three models for all gap deletions were the Le and Gascuel (LG) model with discrete gamma distribution and invariable sites (LGGI), the Whelan and Goldman (WAG) model with discrete gamma distribution (WAGG), and the WAG model with discrete gamma distribution and invariable sites (WAGGI) [124,125]. The Nearest-Neighbor-Interchange (NNI) maximum likelihood method was utilized, with the initial tree made by employment of the default neighbor joining (NJ/BioNJ) method. A total of nine trees were produced (3 models × 3 gap deletions) with 1000 bootstrap replications each. Dendroscope v3.5.9 [126] was used to visualize the phylogenetic trees, and the branching consistency of each tree with its corresponding consensus tree was checked. The best model and gap deletion were picked from the trees which showed similar branching and bootstrap values to their consensus trees.

### 3.4. Motif Analysis

Motif analysis was carried out using Multiple Expectation Maximisation for Motif Elicitation (MEME) v4.11 [88], with a motif width of 3–20 amino acids, to identify conserved patterns in potentially important functional regions. A maximum of 20 motifs was searched. Overlapping motifs were identified using motif pairwise correlations calculated by the Motif Alignment and Search Tool (MAST) [127]. High correlations observed above 0.6 indicated motif redundancy, and these motifs were consequently not included in the results. Those that showed statistical insignificance (E-value > 0.05) were also removed from the results. An in-house MATLAB script which calculates the frequency of motif occurrence across the sequences was used to produce a heat map displaying motif conservation [121,128].

### 3.5. Homology Modelling 

LOGACA (PDB ID: 6EKI) was selected as a template and used to model all α-CA proteins except for PmCA and TaCA, whose structures were obtained from the PDB for further analyses. 3D calculations proceeded, utilizing the slow refinement option in automodel by MODELLER v9.20 [129]. The α-CA proteins were modelled as dimers with 100 models being produced per sequence. The first 5 models exhibiting the lowest z-DOPE scores were chosen for further structural validation using Verify3D [93], PROCHECK [92] as well as ProSA [130] servers. Motifs, generated using MEME suite, were mapped onto a representative structure using PyMOL v1.7.2.1 [131], a molecular graphics program. 

### 3.6. Protein–Protein Interface Analysis

To identify interface residues of the multimeric proteins, all models and crystal structures were subjected to interface analysis using five different web servers: Hotregion [132]; Knowledge-based FADE and Contacts (KFC) server [133]; the Protein Interfaces, Surfaces and Assemblies (PDBePISA) web service [96]; PPCheck [134]; and Robetta [94]. The web servers, excluding PDBePISA, were further queried for hotspot residues in the interface. PDBePISA is a web-based tool that implements the graph theory method in analyzing macromolecular complex interfaces, producing information such as the solvent accessible surface areas, BSA and interface residues, as well as inter-subunit hydrogen bonds and salt bridges [96]. Hotregion and KFC make use of machine learning to predict interfacing residues. Robetta identifies interface residues and mutates them to alanine, then calculates the change in binding free energy (ΔΔG_bind_) upon mutation. A change in ΔΔG_bind_ above 1 kcal/mol indicates that mutation of the residue results in destabilization, thus it is regarded as a hotspot residue [94]. PPCheck identifies hotspot residues using alanine scanning as well. Interface residues and hotspot were then selected by consensus approach, as such, for interface residues three out of five, and for hotspot residues three out of four web servers were required to agree. All these residues, along with motifs generated using MEME suite, were mapped onto a representative dimeric structure using PyMOL [131].

Intra-subunit interactions were analyzed using the Protein Interactions Calculator (PIC) web server [95]. It uses standardized cut-offs for a wide range of interactions, including disulfide bridges, hydrophobic, aromatic–aromatic, ionic and cation–Pi interactions as well as hydrogen bonds. In this study, ionic interactions were queried with a cut-off distance of 4 Å. 

### 3.7. Molecular Dynamics Simulations

MD simulations were performed on the dimeric (tetrameric for TaCA) assemblies of the proteins at four different temperatures; 300 K, 363 K, 393 K and 423 K. H++ server [135] was used to protonate the structures at pH 8.00 to mimic the alkaline environment of CO_2_ sequestration. All His residues coordinating Zn^2+^ were manually cross-checked for the correct protonation states. The first two His residues were confirmed to be HIDs, with the δ-nitrogen protonated and the third His residue an HIE, with the ε-nitrogen protonated. Zn^2+^ parameters previously generated by Sanyanga et al. [82] using AmberTools17 [136] and Gaussian09 [137] were inferred onto the α-CA structures. The AMBER force field ff14SB [138] was utilized. Coordinate and topology files were produced using *tleap* [139] and the system was solvated with a cubic water box of 10 Å. ACPYPE (AnteChamber PYthon Parser interfacE) [140] was utilized for the generation of gmx files for minimization as well as subsequent equilibration and simulation using GROMACS 2016.1 [141] at each of the four temperatures. Steepest descent minimization was performed with an energy step size of 0.01, followed by *NVT*/canonical ensemble equilibration at 300 K, 363 K, 393 K and 423 K in order to stabilize the temperatures. *NPT*/isothermal-isobaric equilibration was performed at the temperature of the corresponding *NVT,* and a pressure of 1 atm followed by 50 ns MD simulations at the same temperature. All calculations were performed at Centre for High Performance Computing (CHPC) clusters in Cape Town (SA). All simulations ran with a time step of 2 fs and coordinates were saved every 10 ps during the simulation. Root mean square fluctuation (RMSF) values were calculated for the residues of each complex. The radius of gyration (R_g_)was calculated for the individual chains of each complex, and for the individual catalytic pockets. Hydrogen bond interactions across the multimers were identified using the hbond command in *cpptraj* [104] from AmberTools17 with a donor-acceptor distance cut-off of 3.5 Å. Define Secondary Structure of Protein (DSSP) analysis [111] of interface residues was performed using the secstruct command, also in *cpptraj.*

### 3.8. Dynamic Residue Network Analysis

*Betweenness centrality* (*BC*) for each residue in the multimeric α-Cas and C_β_ atoms (C_α_ for Gly), was calculated by use of the MD-TASK tool [50]. *BC* measures the usage of a node by calculating how often the shortest paths from all other nodes pass through it. The script, *calc_network.py*, in MD-TASK was used for the calculation of *BC* over the last 20 ns of the trajectories with a threshold of 6.7 Å, followed by the calculation of average *BC* using the *avg_network.py* script over MD trajectories. Average *BC* for each protein was normalized, scaling all values into the range 0–1. High average *BC* values directly translate to high residue usage, substantiating its importance in protein communication.

### 3.9. Dynamic cross Correlation

Dynamic cross correlation (DCC) analysis was implemented for the C_α_ atoms in each multimeric protein. Matrices were produced by MD-TASK software using the *calc_correlation.py* script over the entire 50 ns trajectories using the following equation:

(1) $$Cij\;=\;\frac{\langle \Delta \;r_{i}.\Delta \;r_{j}\rangle }{{\left(\langle \Delta r_{i}^{2}\Delta r_{j}^{2}\rangle \right)}^{\frac{1}{2}}}$$

Correlation matrix heatmaps for each trajectory were plotted using Mathematica v11.3 (Wolfram Research Inc.: Champaign, IL, USA) [142].

## 4. Conclusions

The urgency of the discovery of thermostable carbon dioxide sequestration agents motivated the present study of α-CAs from bacteria with hydrothermal vent origins. With most studies focusing on the monomeric forms of the enzymes, this study reflected markedly on the importance of the protein-protein interactions formed by the multimeric proteins for their function, as well as stability, at high temperatures. 

Various computational techniques were successfully employed in the retrieval and analysis of a total of 15 CAs, including LOGACA, PmCA and TaCA, which had predetermined structures. The other structures were calculated using homology modelling and several validation programs deemed the dimeric structures to be of good quality. Sequence alignments and motif analysis revealed high levels of conservation amongst the CAs with all but two (*Geothermobacter* CAs) showing the presence of signal peptides. Hotspot and other interface residues were identified for the multimeric structures by various web servers. Importance of these residues in protein function was further fortified by their presence in the group of residues showing high usage via average *BC* calculations over MD simulation trajectories at 300 K. The detection of important intra-subunit hydrogen bond networks was also achieved. The unique network identified in NtCA, involving a combination of high communication residues, hotspot residues as well as Cys205 (NtCA numbering) which forms an intra-subunit disulfide bond, is proposed to have a significant contribution towards its thermostability.

Residue composition of the interface motifs proved crucial to protein thermostability after investigating behavior of the interface residues using RMSF, DSSP and average *BC* analyses of simulations at 300 K, 363 K, 393 K and 423 K. CmCA, LOGACA, PhCA and PmCA, which contained hydrophobic residues shielding the interface core from solvent accessibility, demonstrated lower fluctuations, and consequently higher thermostability properties. This was also accompanied by consistency in interface hydrogen bonds at high temperatures. All proteins showed high correlation between chains at one or more temperatures, further fortifying the importance of interface residues. TaCA portrayed a slightly different behavior compared to the rest of the CAs, with further confirmation of the two disulfide bonds in the tetramer interface as the main source of its thermostability. The importance of the catalytic cavity was also considered and monitored using R_g_ analysis, with most proteins maintaining compactness of the cavities at high temperatures. It is also worth noting that, for all CAs, asymmetrical behavior was observed for monomers of the same protein throughout the analyses.

Ultimately, a consensus of all the methods utilized in this study revealed NtCA, besides those previously characterized, as the CA with the highest potential for thermostability, showing low protein residue fluctuations and high synchronized motions, as well as the maintenance of chain and cavity compactness at high temperatures. Runner-up candidates include GEprmCA, PhCA, SlCA and VaCA1. Overall, this study showed the importance of simulating these proteins in their multimeric assemblies and serves as a basis for in vitro analysis of these α-CAs to prove their usefulness in the biotechnology industry as thermostable CO_2_ sequestration agents. It also revealed important features of the CAs responsible for thermostability, and thus serves as a basis for engineering of less thermostable dimeric CAs.

## Figures and Tables

**Table 1:** Alpha-carbonic anhydrase **(**α-CA) proteins from hydrothermal vent bacteria with taxonomic classifications.

CA	Organism	Taxonomic Classification			
		Class	Family	Genus	Reference
CmCA	*Caminibacter* *mediatlanticus*	Campylobacteria	Nautiliaceae	*Caminibacter*	[58]
GEprmCA	*Geothermobacter* sp. EPR-M	Deltaproteobacteria	Geobacteraceae	*Geothermobacter*	[63]
GHr1CA	*Geothermobacter* sp. HR-1	Deltaproteobacteria	Geobacteraceae	*Geothermobacter*	[63]
HtCA	*Hydrogenimonas thermophila*	Campylobacteria	Hydrogenimonaceae	*Hydrogenimonas*	[58]
LOGACA	Hydrothermal vent metagenome	---	---	---	---
NtCA	*Nitratiruptor* *tergarcus*	Campylobacteria	Nitratiruptoraceae	*Nitratiruptor*	[58]
PhCA	*Persephonella hydrogeniphila*	Aquificacea	Hydrogenothermaceae	*Persephonella*	[63]
PmCA	*Persephonella* *marina*	Aquificacea	Hydrogenothermaceae	*Persephonella*	[63]
SlCA	*Sulfurovum* *lithotrophicum*	Campylobacteria	Sulfurovaceae	*Sulfurovum*	[58]
SNbcCA	*Sulfurovum* sp. NBC37-1	Campylobacteria	Sulfurovaceae	*Sulfurovum*	[58]
SrCA	*Sulfurovum* *riftiae*	Campylobacteria	Sulfurovaceae	*Sulfurovum*	[58]
TaCA	*Thermovibrio ammonificans*	Aquificacea	Desulfurobacteriaceae	*Thermovibrio*	[63]
VaCA1 VaCA2	*Vibrio antiquarius*	Gammaproteobacteria	Vibrionaceae	*Vibrio*	[63]
VdCA	*Vibrio diabolicus*	Gammaproteobacteria	Vibrionaceae	*Vibrio*	[63]

**Table 2:** Motifs containing known functional residues in α-CAs. Motif residues displayed are from *Persephonella marina* (PmCA), and those in bold and underlined have been identified as functionally important.

Motif	E-Value	Residues	Function
1	1.5 × 10^−251^	YY**RYS**GS**LTT**PP**C**S**EGVRW**I	**Cys197**—intra-subunit disulfide Cys [29,34,74,75,76] **Leu192, Val201 & Trp203**—CO_2_ binding pocket residues [29,37,75] **Thr193 & Thr194**—proton shuttling residues [29,34] **Arg187, Tyr188, Ser189, Glu199, Gly200, Val201 & Arg202**—interface residues (present study)
2	9.9 × 10^−180^	QF**H**F**H**APSEHKLKGQHYPFE	**His107 & His109**—Zn^2+^ coordinating residues [30,31,32]
3	1.6 × 10^−146^	MGGDTNRPVQPL**NARM**IME	**Asn236, Ala237, Arg238, Met239 & Met241**—interface residues (present study)
4	7.8 × 10^−146^	A**H**F**V**HADKHG**N**LA**V**IGVFFK	**His126**—Zn^2+^ coordinating residues [30,31,32] **Val128 & Val138**—CO_2_ binding pocket residing residues [29,34,37,75] **Asn135**—interface residue (present study)
5	2.4 × 10^−119^	**Y**HGEHGPEHWGDLK**D**EY**IMC**	**Tyr25**—proton shuttling residue [29,34] **Asp39, Ile42 and Met43**—interface residues (present study) **Cys44**—intra-subunit disulfide Cys residue [29,34,74,75,76]
6	3.3 × 10^−110^	N**N**G**H**TI**K**VSYEPGSYIVV**D**G	**Asn80, His82 & Lys85**—proton shuttling residues [29,34,77] **Asp97**—interface residues (present study)
7	7.2 × 10^−77^	**KN**QSPVDIN**R**IV**DAKL**KPIK	**Lys48, Asn49, Arg57, Val59, Asp60, Ala61, Lys62 & Leu63**—interface residues (present study)

**Table 3:** The top 5% residues showing the highest average *BC* values. All residues with known functions are in bold and interface residues are italicized.

CA	Residues
CmCA	**Chain A**: ***Asn53***^h^, Asn122, Glu132, Ile144, **Val146**^c^, Ala148, ***Asn197***, ***Glu207***^h^, ***Gly208***, ***Val209***^c^, **Trp211**^c^, Ile247
	**Chain B**: ***Asn53***^h^ Ile57, Asn122, Glu132, **Val146** ^c^, Ala148, ***Phe196***, ***Asn197***, Ser199, ***Glu207*** ^h^, ***Val209*** ^c^
GEprmCA	**Chain A**: ***Arg48***, Asn114, Glu124, ***Asn135***, **Val138**^c^, Ser140, ***Phe188***, ***Val201***^c^, ***Ile202***, **Trp203**^c^, ***Arg238***
	**Chain B**: ***Arg48***, Val53, Ile96, Asn114, Glu124, ***Asn135***, **Val138 ^c^**, Ser140, ***Val201***^c^, **Trp203**^c^, ***Ala237***, ***Arg238***
GHr1CA	**Chain A**: ***Asn38***^h^, Glu113, ***Asn124***, **Val127**^c^, Ser129, ***Phe177***, ***Asn178***, ***Gln188***^h^, **Trp192**^c^, ***Arg227***
	**Chain B**: ***Asn38***^h^, Val42, Asn103, Glu113, **Val127**^c^, Ser129, ***Asn178***, ***Gly189***, ***Val190***^c^, **Trp192**^c^, Leu224, ***Ala226***, ***Arg227***^h^
HtCA	**Chain A**: ***Asn54***^h^, Glu130, **His132**^z^, **Val144**^c^, ***Asn196***, Ser198, **Thr200**^p^, ***Glu206***^h^, ***Val208***^c^, **Trp210**^c^
	**Chain B**: ***Asn54***^h^, Glu130, **His132**^z^, **Val144**^c^, Ala146, ***Asn196***, Ser198, ***Glu206***^h^, ***Val208***^c^, **Trp210**^c^, ***Arg245***^h^, Ile247
LOGACA	**Chain A**: ***Asn59***^h^, ***Asp107***, **His136**^z^, **Val148**^c^, ***Ser198***, Ser200, **Cys206**^d^, ***Gly209****,****Val210***^c^, ***Ala246***^h^, ***Arg247***
	**Chain B**: ***Asn59****^h^*, Val63, His124, Glu134, **Val148**^c^, ***Tyr197***, Ser200, ***Gly209***, ***Val210***^c^*,***Trp212**^c^, ***Arg247***
NtCA	**Chain A**: Gln58, Val61, Asn122, Glu132, *Glu143*, **Val146**^c^, Ala148, ***Tyr196***, ***Val209***^c^, **Trp211**^c^, ***Arg245***^h^
	**Chain B**: ***Asn57***^h^, Val61, Asn122, ***Glu143***, **Val146**^c^, Ala148, ***Asp197***, ***Val209***^c^, **Trp211**^c^, Leu242, ***His243***, ***Arg245***^h^
PhCA	**Chain A**: ***Asn52***^h^, His117, Glu127, **Val141**^c^, ***Ser192***, Ser194, **Thr196**^p^, **Cys200**^d^, ***Glu202***^h^, ***Val204***^c^, ***Asn239***, ***Arg2410***^h^
	**Chain B**: ***Asn52***^h^, Glu127, **Val141**^c^,***Tyr191***, Ser194, ***Glu202***^h^, ***Gly203***, ***Val204***^c^, ***Arg241***
PmCA	**Chain A**: ***Asn49***^h^, Val53, His114, Glu124, **Val138**^c^, ***Ser189***, Ser191, **Cys197**^d^, ***Val201***^c^, ***Ala237***,
	**Chain B**: ***Asn49***^h^*,* Val53, Ile55, His114, Glu124, **Val138** ^c^, ***Ser189***, Ser191, **Cys197** ^d^,***Glu199*** ^h^, ***Gly200***, ***Val201*** ^c^
SlCA	**Chain A**: Glu129, **Val143**^c^, Ala145, ***Arg192***^h^, ***Phe193***, ***Asn194***, Ser196, ***Val206***^c^, **Trp208**^c^, ***Arg243***^h^
	**Chain B**: ***Asn53***^h^, Ile57, Asn119, Leu128, Glu129, **Val143**^c^, Ala145, ***Asn194***, ***Val206***^c^, **Trp208**^c^, ***Arg243***^h^, ***Val244***^h^, Val245
SNbcCA	**Chain A**: ***Asn53***^h^, Ile57, Glu129, **His131**^z^, Val143 ^c^, Ala145, ***Asn194***, Ser196, ***Glu204***, ***Val206***^c^, **Trp208**^c^, ***Arg243***^h^
	**Chain B**: ***Gly51***, ***Asn53***^h^, Glu129, **Val143**^c^, Ala145, ***Phe193***, ***Asn194***, ***Val206***^c^, **Trp208**^c^, ***Arg243***
SrCA	**Chain A**: ***Asn54***^h^, Ile58, Asn120, Glu130, **Val144**^c^, Ala146, ***Phe193***, ***Glu204***^h^, ***Val206***^c^, **Trp208**^c^, ***Arg243***
	**Chain B**: ***Asn54***^h^, Glu130, **Val144**^c^, Ala146, ***Asn194***, Ser196, ***Glu204***^h^, ***Val206***^c^, **Trp208**^c^, ***Arg243***^h^
TaCA	**Chain A**: Ile58, ***Ser60***, Ala63, ***Val64***^h^, ***Lys65***, ***Cys67***^d^, ***Leu68***, His119, **Val143**^c^, Gly145, Tyr191, ***Arg192***^h^, ***Val206***^c^, Ile209, ***Lys247***
	**Chain B**:***Val64***^h^, ***Lys65***, ***Cys67***^d^, ***Leu68***, Gly145, Tyr190, Tyr191, ***Arg192***^h^, ***Tyr193***, ***Val206***^c^, **Trp208**^c^, Ile209, ***Lys247***
	**Chain C**: Ile58, ***Val64***^h^, ***Lys65***, ***Cys67***^d^, ***Leu68***, His119, Gly145, Tyr191, ***Arg192***^h^, ***Arg243***^h^, ***Lys247***
	**Chain D**: ***Val64***^h^, ***Lys65***, ***Cys67***^d^, ***Leu68***, Tyr190, Tyr191, ***Arg192***^h^, ***Val206***^c^, ***Lys247***
VACA1	**Chain A**: *Thr45*, Ile51, Asn112, Glu122, **His124**^z^, **Val136**^c^, Ser138, ***Phe185***, ***Asn186***, ***Val198***^c^, **Trp200**^c^, ***Ala233***^h^, ***Arg234***^h^
	**Chain B**: Ile51, Asn112, **Val136**^c^, ***Phe185***, ***Asn186***, Ser188, ***Val198***^c^, **Trp200**^c^, ***Ala233***^h^, ***Arg234***^h^
VaCA2	**Chain A: Val135**^c^, Ala137, ***Arg183***^h^, ***Phe184***, ***Gly196***, ***Val197***^c^, **Trp199**^c^, ***Met234***^h^
	**Chain B**: ***Gln45***, ***Asn46***, Ile50, Ile52, Asn111, Glu121, **Val135**^c^, Ala137, ***Phe184***, ***Asn185***, **Trp199**^c^, ***Asn231***, ***Ala23***^h^, ***Arg233***, ***Met234***^h^, Val235
VdCA	**Chain A**: Ile50, Asn111, Glu121, **Val135**^c^, Ala137, ***Phe184***, ***Asn185***, ***Val197***^c^, **Trp199**^c^, ***Ala232***^h^, ***Arg233***^h^
	**Chain B**: ***Asn46***^h^, Ile50, Asn111, Glu121, **Val135**^c^, Ala137, ***Phe184***, ***Asn185***, ***Val197***^c^, **Trp199**^c^, ***Arg233***^h^

^h^ Hotspot residues; ^z^ Active site His residue; ^c^ CO_2_ binding pocket residue; ^d^ Cys–Cys disulfide bond residue; ^p^ proton shuttling residues.

## Supplementary Materials

Supplementary materials can be found at https://www.mdpi.com/1422-0067/21/21/8066/s1.

## Abbreviations

*BC*	*Betweenness centrality*
CA	Carbonic anhydrase
CmCA	*Caminibacter mediatlanticus* carbonic anhydrase
DCC	Dynamic cross correlation
DRN	Dynamic residue network
DSSP	Define Secondary Structure of Proteins
GEprmCA	*Geothermobacter* sp. EPR-M carbonic anhydrase
GHr1CA	*Geothermobacter* sp. HR-1 carbonic anhydrase
HtCA	*Hydrogenimonas thermophila* carbonic anhydrase
LOGACA	Logatchev hydrothermal field carbonic anhydrase
MSA	Multiple sequence alignment
NtCA	*Nitratiruptor tergarcus* carbonic anhydrase
PhCA	*Persephonella hydrogeniphila* carbonic anhydrase
PmCA	*Persephonella marina* carbonic anhydrase
RMSF	Root mean square fluctuation
R_g_	Radius of gyration
SI	Sequence identity
SlCA	*Sulfurovum lithotrophicum* carbonic anhydrase
SNbcCA	*Sulfurovum* sp. NBC37-1 carbonic anhydrase
SrCA	*Sulfurovum riftiae* carbonic anhydrase
VaCA1	*Vibrio antiquarius* carbonic anhydrase 1
VaCA2	*Vibrio antiquarius* carbonic anhydrase 2
VdCA	*Vibrio diabolicus* carbonic anhydrase
