# Supplementary material for: Alpha-Carbonic Anhydrases from Hydrothermal Vent Sources as Potential Carbon Dioxide Sequestration Agents: In Silico Sequence, Structure and Dynamics Analyses
Source: Int J Mol Sci. 2020 Oct 29;21(21):8066. doi: 10.3390/ijms21218066 (PMC7662607; doi:10.3390/ijms21218066)
Supplement: Supplementary file 1 [file ijms-21-08066-s001.pdf]

Supplementary data: Alpha-carbonic anhydrases from hydrothermal vent sources as potential carbon dioxide sequestration agents: *In silico* sequence, structure and dynamics analyses of α-carbonic anhydrases

Colleen Varaidzo Manyumwa[1], Reza Zolfaghari Emameh[2] and Özlem Tastan Bishop[1]*

[1] Research Unit in Bioinformatics (RUBi), Department of Biochemistry and Microbiology, Rhodes University, Grahamstown, 6140, South Africa; colleen.manyumwa06@gmail.com; O.TastanBishop@ru.ac.za

[2] Department of Energy and Environmental Biotechnology, National Institute of Genetic Engineering and Biotechnology (NIGEB), 14965/161, Tehran, Iran; zolfaghari@nigeb.ac.ir

*Correspondence: O.TastanBishop@ru.ac.za; Tel: +27-46-603-8072; Fax: +27-46-603-7576

Table S1: List of NCBI accession numbers for each α-CA selected for the study with initial query sequences highlighted in blue.

Organism	CA abbreviation	Accession number	Sequence length	Query sequence	Query cover	Sequence identity	E-value
Caminibacter mediatlanticus	CmCA	WP_007474387.1	249 aa	*C. mediatlanticus*	100%	100%	$1e^{-156}$
Persephonella marina	PmCA	WP_015898908.1	243 aa	*P. marina*	100%	100%	$2e^{-172}$
Thermovibrio ammonificans	TaCA	WP_013538320.1	247 aa	*T. ammonificans*	100%	100%	$4e^{-160}$
Geothermobacter sp. EPR-M	GEprmCA	WP_139800730	231 aa	*P. marina*	90%	52.94%	$2e^{-149}$
Geothermobacter sp. HR-1	GHr1CA	WP_103115889.1	246 aa	*P. marina*	90%	51.58%	$2e^{-150}$
Hydrogenimonas thermophila	HtCA	WP_092911809.1	249 aa	*T. ammonificans*	100%	48.80%	$2e^{-81}$
–	LOGACA	PDB: 6EKI_A	226 aa	*P. marina*	91%	81%	$2e^{-134}$
Nitratiruptor tergarcus	NtCA	WP_084275285.1	249 aa	*C. mediatlanticus*	100%	50.99%	$4e^{-137}$
Persephonella hydrogeniphila	PhCA	WP_096999253.1	247 aa	*P. marina*	99%	75.00%	$5e^{-161}$
Sulfurovum lithotrophicum	SlCA	WP_052746102.1	248 aa	*T. ammonificans*	100%	51.21%	$5e^{-151}$
Sulfurovum sp. NBC37-1	SNbcCA	WP_011980858.1	248 aa	*T. ammonificans*	100%	50.40%	$3e^{-150}$
Sulfurovum riftiae	SrCA	WP_067332141.1	248 aa	*T. ammonificans*	100%	50.20%	$9e^{-150}$
Vibrio antiquarius(1)	VaCA1	WP_006741278.1	239 aa	*P. marina*	99%	44.03%	$4e^{-88}$
Vibrio antiquarius(2)	VaCA2	WP_006741954.1	239 aa	*P. marina*	100%	48.56%	$1e^{-146}$
Vibrio diabolicus	VdCA	WP_104970467.1	239 aa	*P. marina*	100%	47.74%	$3e^{-146}$

Table S2: The residue numbers for important functional residues in the α-CAs of each organism.

Carbonic anhydrase	Active site residues			Proton transfer residues						CO_2 binding pocket residues					Disulfide bond Cys residues	
CmCA	His115	His117	His134	His90	Tyr29	Asn88	Lys93	Thr201	Thr202	Val136	Val146	Leu200	Val209	Trp211	Cys48	Cys205
GEprmCA	His107	His109	His126	His82	Tyr25	Asn80	Gln85	Thr193	Thr194	Val128	Val138	Leu192	Val201	Trp203	Cys44	Cys197
GHr1CA	His96	His98	His115	His71	Tyr14	Asn69	Gln74	Thr182	Thr183	Val117	Val127	Leu181	Val190	Trp192	Cys33	Cys186
HtCA	His113	His115	His132	His88	Tyr30	Lys86	Lys91	Thr200	Thr201	Val134	Val144	Leu199	Val208	Trp210	Cys49	Cys204
LOGACA	His117	His119	His136	His92	Tyr35	Lys90	Lys95	Thr202	Thr203	Val138	Val148	Leu201	Val210	Trp212	Cys54	Cys206
NtCA	His115	His117	His134	His90	Tyr33	Asn88	Lys93	Thr201	Thr202	Val136	Val146	Phe200	Val209	Trp211	Cys52	Cys205
PmCA	His107	His109	His126	His82	Tyr25	Asn80	Lys85	Thr193	Thr194	Val128	Val138	Leu192	Val201	Trp203	Cys44	Cys197
PhCA	His110	His112	His129	His85	Tyr28	Asn83	Lys88	Thr196	Thr197	Val131	Val141	Leu195	Val204	Trp206	Cys47	Cys200
SlCA	His112	His114	His131	His87	Tyr29	Asn85	Gln90	Thr198	Thr199	Val133	Val143	Leu197	Val206	Trp208	Cys48	Cys202
SNbcCA	His112	His114	His131	His87	Tyr29	Asn85	Gln90	Thr198	Thr199	Val133	Val143	Leu197	Val206	Trp208	Cys48	Cys202
SrCA	His113	His115	His132	His88	Tyr30	Asn86	Gln91	Thr198	Thr199	Val134	Val144	Leu197	Val206	Trp208	Cys49	Cys202
TaCA	His112	His114	His131	His87	Tyr28	Asn85	Lys90	Thr198	Thr199	Val133	Val143	Leu197	Val206	Trp208	Cys47	Cys202
VaCA1	His105	His107	His124	His80	Tyr25	Asn78	Gln83	Thr190	Thr191	Val126	Val136	Leu189	Val198	Trp200	Cys42	Cys194
VaCA2	His104	His106	His123	His79	Tyr25	Asn77	Gln82	Thr189	Thr190	Val125	Val135	Leu188	Val197	Trp199	Cys41	Cys193
VdCA	His104	His106	His123	His79	Tyr25	Asn77	Gln82	Thr189	Thr190	Val125	Val135	Leu188	Val197	Trp199	Cys41	Cys193

Table S3: Signal peptide cleavage sites prognosticated by various signal peptide predictor programs. The last column shows the first residue number in the modelled chain after removing unavailable structural information from the template.

Carbonic anhydrase	SignalBLAST cleavage site	Phobius cleavage site	Starting aa in model
CmCA	25	20	26
GEprmCA	–	–	22
GHr1CA	–	–	11
HtCA	21	20	27
NtCA	19	21	30
PhCA	23	20	25
SlCA	23	22	26
SNbcCA	23	22	26
SrCA	23	20	27
VaCA1	18	20	22
VaCA2	22	20	22
VdCA	22	20	22

Table S4: Motifs generated by MEME, their positions in the α-CAs and their respective E-values.

Organism	Motif number and E-value													
	**1**	**2**	**3**	**4**	**5**	**6**	**7**	**8**	**9**	**10**	**11**	**12**	**13**	**14**
	$1.8e^{-251}$	$9.9e^{-180}$	$1.6e^{-146}$	$7.8e^{-146}$	$2.4e^{-119}$	$3.3e^{-110}$	$7.2e^{-77}$	$5.4e^{-75}$	$1.6e^{-66}$	$7.7e^{-28}$	$4.6e^{-13}$	$1.0e^{-6}$	$1.8e^{-4}$	$6.9e^{-4}$
CmCA	193-212	113-132	231-249	133-152	29-48	87-106	52-71	213-228	153-172	177-192	1-16	–	22-28	–
GEprmCA	185-204	105-124	224-242	125-144	25-44	79-98	48-67	205-220	145-164	169-184	–	100-104	18-24	–
GHr1CA	174-193	94-113	213-231	114-133	14-33	68-87	37-56	194-209	134-153	158-173	–	89-93	7-13	–
HtCA	192-211	111-130	231-249	131-150	30-49	85-104	53-72	212-227	151-170	176-191	1-16	106-110	23-29	–
LOGACA	167-186	88-107	206-244	108-127	8-27	62-81	31-50	187-202	128-147	151-166	–	83-87	1-7	–
NtCA	193-212	113-132	231-249	133-152	33-52	87-106	56-75	213-228	153-172	177-192	1-16	–	26-32	–
PhCA	188-207	108-127	227-245	128-147	28-47	82-101	51-70	208-223	148-167	172-187	1-16	103-107	21-27	–
PmCA	185-204	105-124	224-242	125-144	25-44	79-98	48-67	205-220	145-164	169-184	1-16	100-104	18-24	–
SlCA	190-209	110-129	229-247	130-149	29-48	84-103	52-71	210-225	150-169	174-189	1-16	105-109	22-28	72-83
SNbcCA	190-209	110-129	229-247	130-149	29-48	84-103	52-71	210-225	150-169	174-189	1-16	105-109	22-28	72-83
SrCA	190-209	111-130	229-247	131-150	30-49	85-104	53-72	210-225	151-170	174-189	1-16	106-110	23-29	73-84
TaCA	190-209	110-129	229-247	130-149	28-47	84-103	51-70	210-225	150-169	174-189	1-16	105-109	21-27	–
VaCA1	182-201	103-122	220-238	123-142	26-45	77-96	46-65	202-217	143-162	166-181	1-16	98-102	18-24	–
VaCA2	181-200	102-121	219-237	122-141	25-44	76-95	45-64	201-216	142-161	165-180	1-16	97-101	18-24	–
VdCA	181-200	102-121	219-237	122-141	25-44	76-95	45-64	201-216	142-161	165-180	1-16	97-101	18-24	–

Table S5: Template coverage, sequence identity and model validation for α-CA sequences.

Organism	Template PDB ID	Sequence identity (%)	Sequence coverage (%)	z-Dope score	Verify3D (%)	ProSA (*z-score*)	PROCHECK	
							Most favoured region (%)	Disallowed region(%)
CmCA	LOGACA	49	89	-1.11	91	-7.76	89.6	0
GEprmCA		52	90	-1.04	88.2	-6.55	91.8	0
GHr1CA		51	95	-1.05	95.2	-6.32	92.7	0
HtCA		49	88	-0.82	80.7	-7.46	87.6	1.3
NtCA		52	88	-1.08	83.4	-6.73	90.9	0.3
PhCA		84	89	-1.42	96.4	-7.2	91.4	0
SlCA		48	88	-1.04	89	-6.59	89.9	0
SNbcCA		47	88	-1.0	85.2	-6.57	89.4	0.5
SrCA		49	88	-1.13	92.1	-6.85	89.5	0
VaCA1		50	90	-1.11	91.7	-6.65	89.3	0.8
VaCA2		50	91	-0.79	89.7	-6.29	90.2	0.8
VdCA		50	91	-0.81	90.1	-6.32	89	0

Table S6: Residues confirmed by at least three of the interface prediction servers to be found in the interface of α-CAs in this study. Hotspot residues are shown in bold.

CA	Interface residues
CmCA	**Chain A:** Q46, M47, I50, G51, K52, **N53**, T64, Q65, **F67**, N68, T69, N70, L71, K72, D105, N143, R195, N197, G198, S206, **E207**, G208, V209, R210, N243, A244, R245, **V246**, **L248**<br>**Chain B:** Q46, M47, I50, G51, K52, **N53**, S63, T64, Q65, **F67**, N68, T69, N70, L71, K72, D105, N143, R195, F171, N197, S206, **E207**, G208, V209, R210, N243, A244, R245, V246, L248
GEprmCA	**Chain A:** I43, S46, G47, R48, **N49 V59**, D60, A61, E62, L63, P64, D97, **R187**, F188, N189, Q199, G200, V201, I202, N236, **A237**, R238, L239, **L241**<br>**Chain B:** I43, S46, R48, **N49**, V59, D60, A61, E62, L63, P64, D97, R99, N135, **R187**, F188, N189, S198, **Q199**, G200, V201, I202, L235, N236, A237, R238, L239, **L241**
GHr1CA	**Chain A:** I32, S35, G36, R37, **N38**, R45, V48, D49, A50, E51, L52, P53, D86, N124, R176, F177, N178, S187, **Q188**, G189, V190, I191, N225, **A226**, R227, L228, **L230**<br>**Chain B:** I32, S35, G36, R37, **N38**, **V48**, D49, A50, E51, L52, P53, D86, R88, N124, R176, F177, N178, S187, **Q188**, G189, V190, I191, N225, A226, **R227**, L228, **L230**
HtCA	**Chain A:** E47, M48, I51, G52, K53, **N54**, T62, Q63, **Y66**, D67, V68, D69, L70, E71, D103, Q141, R194, Y195, N196, T205, **E206**, G207, V208, R209, N243, A244, R245, V246, L248<br>**Chain B:** E47, M48, I51, G52, K53, **N54**, T62, **Y66**, D67, V68, D69, L70, E71, D103, Q141, R194, Y195, N196, G197, **E206**, G207, V208, **R209**, I242, N243, A244, **R245**, V246, L248
LOGACA	**Chain A:** I52, M53, I56, G57, K58, N59, R67, **V69**, E70, A71, E72, L73, D107, I109, N145, R196, Y197, S198, G199, S207, E208, G209, V210, R211, N245, **A246**, R247, **M248**, **M250**, D253<br>**Chain B:** N49, I52, M53, I56, G57, K58, N59, R67, **V69**, E70, A71, E72, L73, D107, I109, N145, R196, Y197, S198, G199, S207, **E208**, G209, V210, **R211**, N245, **A246**, R247, M248, M250, D253
NtCA	**Chain A:** P47, R50, M51, F54, G55, **V56**, **N57**, R65, I67, E68, A69, K70, L71, P72, D105, I107, E143, R195, Y196, D197, **E207**, G208, V209, **R210**, H243, A244, **R245**, V246, L248<br>**Chain B:** P47, R50, M51, F54, G55, **V56**, **N57**, R65, I67, E68, A69, K70, L71, P72, D105, I107, E143, R195, Y196, D197, T206, E207, G208, V209, **R210**, H243, A244, **R245**, V246, L248
PhCA	**Chain A:** I45, M46, I49, G50, K51, **N52**, R60, A62, E63, A64, D65, L66, D100, I102,N138, R190, Y191, S192, **E202**, G203, V204, **R205**, N239, A240, **R241**, M242, I243, L244, D247<br>**Chain B:** D42, I45, M46, I49, G50, K51, **N52**, R60, A62, E63, A64, D65, L66, D100, G101, I102, N138, R190, Y191, S192, S201, **E202**, G203, V204, R205, N239, A240, R241, M242, I243, L244, D247
PmCA	**Chain A:** I42, M43, I46, G47, K48, **N49**, R57, V59, D60, A71, K62, L63, D97, I99, N135, R187, Y188, S189, E199, G200, V201, **R202**, N236, **A237**, **R238**, M239, M241<br>**Chain B:** D39, I42, M43, I46, G47, K48, **N49**, R57, **V59**, D60, A71, K62, L63, D97, I99, N135, R187, Y188, S189, S198, **E199**, G200, V201, R202, N236, **A237**, R238, M239, M241
SlCA	**Chain A:** H46, M47, E50, G51, L52, **N53**, H61, S62, S64, D65, P66, N67, H68, D102, N140, R192, F193, N194, E204, G205, V206, R207, D241, **A242**, **R243**, V244, V246<br>**Chain B:** E43, H46, M47, E50, G51, L52, **N53**, H61, S62, S64, D65, P66, N67, H68, D102, N140, **R192**, F193, N194, **E204**, G205, V206, R207, D241, **A242**, **R243**, V244, V246
SNbcCA	**Chain A:** H46, M47, E50, G51, L52, **N53**, H61, S62, S64, D65, P66, N67, H68, D102, D140, R192, F193, N194, G195, T203, E204, G205, V206, R207, D241, **A242**, **R243**, V244, V246<br>**Chain B:** E43, H46, M47, E50, L52, **N53**, H61, S62, S64, D65, P66, N67, H68, D102, D140, R192, F193, N194, T203, E204, G205, V206, R207, D241, **A242**, R243, V244, V246
SrCA	**Chain A:** H47, Q48, D51, L53, **N54**, H62, S63, I64, T65, D66, P67, D68, H69, K70, D103, D141, **R192**, F193, N194, T203, **E204**, G205, V206, R207, D241, **A242**, R243, V244, V246<br>**Chain B:** H47, Q48, D51, G52, L53, **N54**, H62, T65, D66, P67, D68, H69, K70, D103, D141, R192, F193, N194, T203, **E204**, G205, V206, R207, D241, **A242**, **R243**, V244, V246
TaCA	**Chain A:**L45, M46, I49, G50, K51, **N52**, **V64**, K65, A66, C67, L68, D102, N140, D189, R192, Y193, S194, G195, S203, E204, G205, V206, R207, N241, **A242**, **R243**, K244, **M246**, K247<br>**Chain B:**L45, M46, I49, G50, K51, **N52**, **V64**, K65, A66, C67, L68, D102, D189, **R192**, Y193, S194, S203, E204, G205, V206, R207, N241, A242, R243, K244, **M246**, K247<br>**Chain C:** L45, M46, I49, G50, K51, **N52**, **V64**, K65, A66, C67, L68, D102, N140, D189, **R192**, Y193, S194, Y168, S203, E204, G205, V206, R207, N241, **A242**, **R243**, K244, **M246**, K247<br>**Chain D:** L45, M46, I49, K51, **N52**, **V64**, K65, A66, C67, L68, D102, D189, **R192**, Y193, S194, S203, E204, G205, V206, R207, N241, A242, R243, K244, **M246**, K247
VaCA1	**Chain A:** K40, D41, T45, **K46**, **N47**, N55, A57, D58, A59, K60, L61, D95, E133, R184, N186, S195, **E196**, G197, V198, R199, **N232**, **A233**, R234, V235, L237, N239<br>**Chain B:** K40, T44, T45, K46, **N47**, N55, A57, D58, A59, K60, L61, D95, E133, R184, F185, N186, S195, **E196**, G197, V198, R199, N232, **A233**, R234, V235, **L237**, N239
VaCA2	**Chain A:** K43, G44, Q45, **N46**, S54, A56, E57, A58, K59, L60, D94, H132, R183, F184, N185, S194, **E195**, G196, V197, R198, N231, **A232**, R233, **M234**, L236, H239<br>**Chain B:** **Q45**, **N46**, S54, A56, E57, A58, K59, L60, D94, H132, R183, F184, **N185**, S194, **E195**, G196, V197, R198, N231, **A232**, R233, **M234**, **L236**
VdCA	**Chain A:** S39, K43, G44, **Q45**, **N46**, S54, A56, E57, A58, K59, L60, D94, H132, R183, N185, G186, S194, **E195**, G196, V197, R198, N231, **A232**, **R233**, M234, L236<br>**Chain B:** S39, K43, Q45, **N46**, S54, A56, E57, A58, K59, L60, D94, H132, R183, F184, N185, S194, **E195**, G196, V197, R198, N231, **A232**, R233, **M234**, L236

Table S7: Interface characteristics of the multimeric structures of α-CAs.

Organism	Hydrogen bonds		Salt bridges		Buried surface area/ Å^2	Total surface area/ Å^2	% BSA
	PDBePISA	PIC	PDBePISA	PIC	PDBePISA		
CmCA	13	19	–	–	2095	22277	9.4
GEprmCA	7	16	–	–	1670	21424	7.8
GHr1CA	8	22	–	–	1677	21425	7.8
HtCA	18	22	–	–	2083	22199	9.4
LOGACA	10	22	8	2	2062	21158	9.5
NtCA	12	24	7	4	1944	21981	8.8
PhCA	11	22	7	2	1967	21334	9.2
PmCA	10	20	8	4	2011	21636	9.3
SlCA	13	22	2	1	1896	21635	8.8
SNbcCA	14	22	2	1	1793	21941	7.9
SrCA	12	13	–	–	1785	21584	8.3
VaCA1	12	21	3	2	1881	21433	8.8
VaCA2	14	24	–	–	1766	21233	8.3
VdCA	12	28	–	–	1725	21059	8.2
TaCA (dimer 1)	8	15	–	1	1820	42781	4.2
TaCA (dimer 2)	8	12	2	2	1784	42781	4.2
TaCA (Tetramerization interface)	12	8	–	–	698	42781	1.6

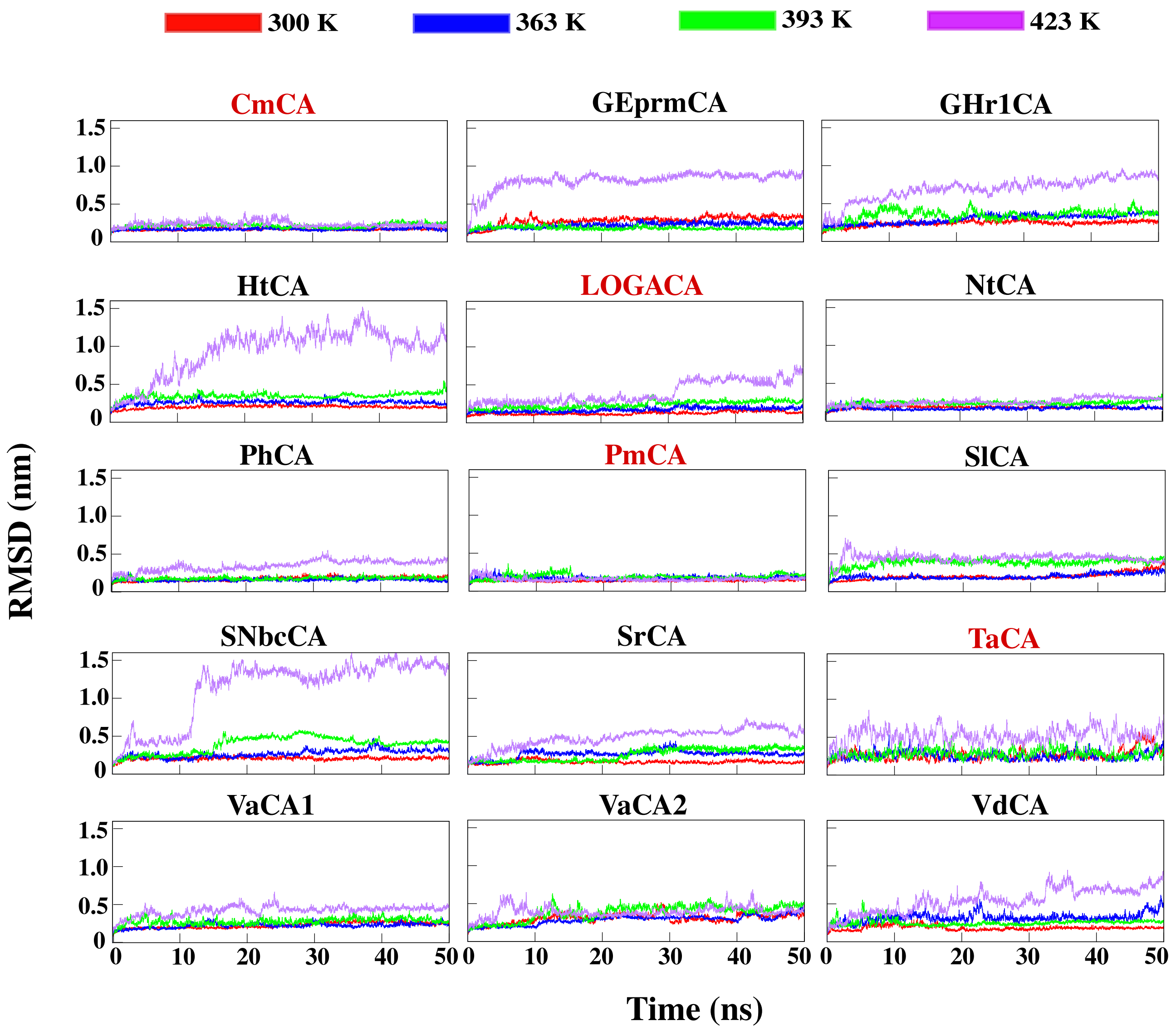

Figure S1: RMSD of the α-CA proteins simulated at 300 K, 363 K, 393 K and 423 K. CmCA, LOGACA, PmCA and TaCA which have been previously characterized, are labelled in red.

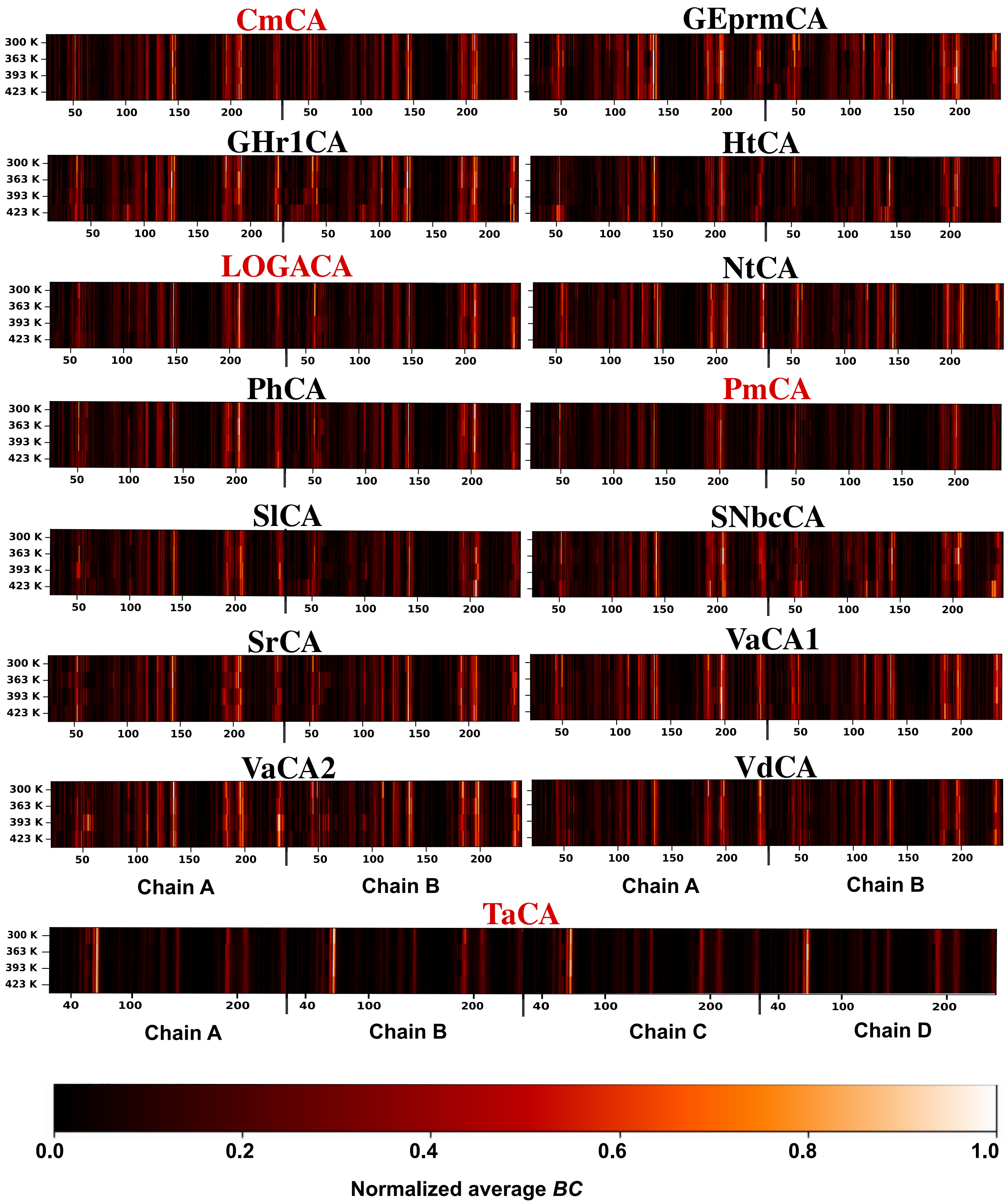

Figure S2: Average *BC*s of residues of the α-CA proteins simulated at 300 K, 363 K, 393 K and 423 K. The average *BC*s were normalized across the temperatures for each protein separately. CmCA, LOGACA, PmCA and TaCA which have been previously characterized, are labelled in red.

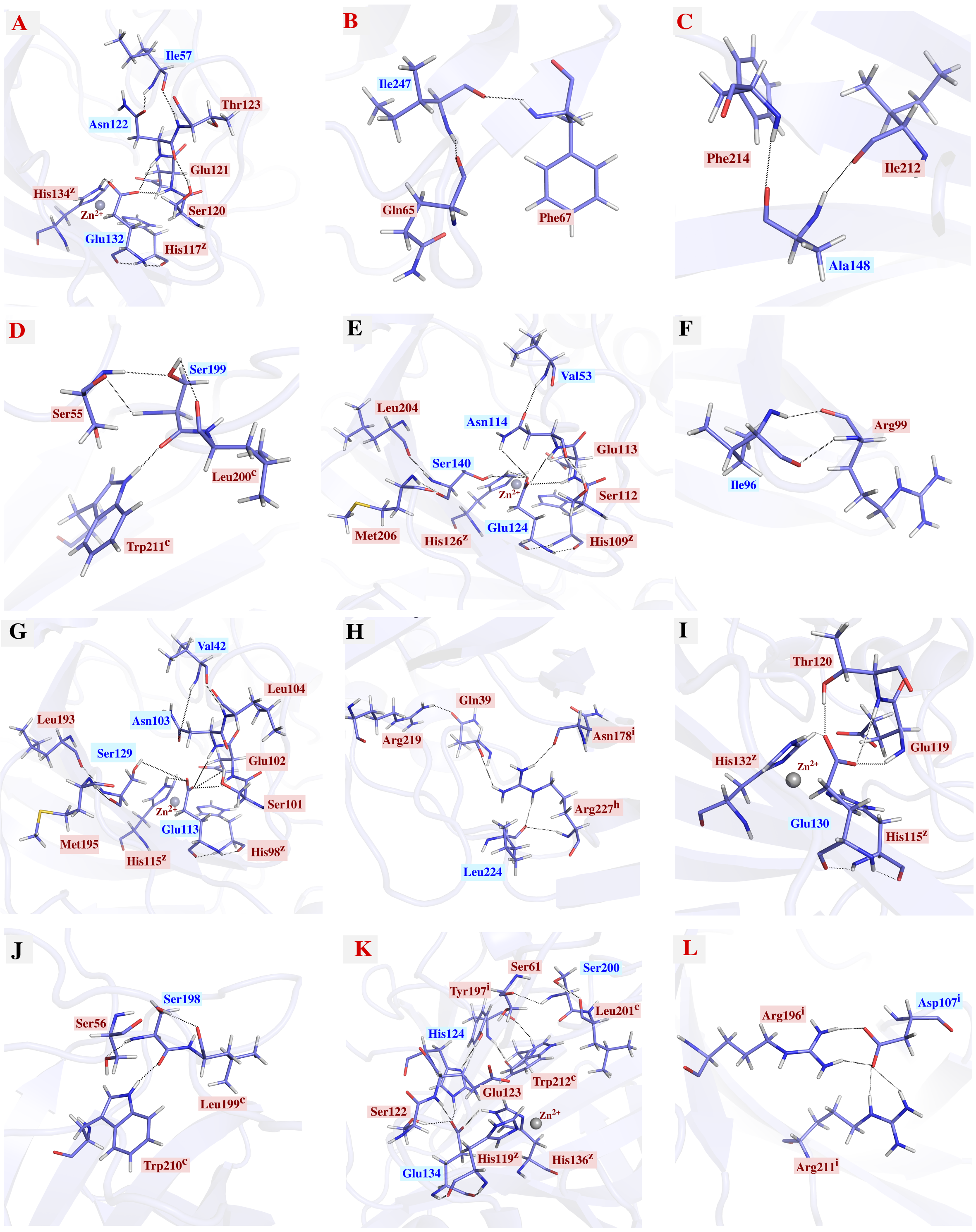

Figure S3: Intra-subunit hydrogen bond networks of CmCA (A-D), GEprmCA (E and F), GHr1CA (G and H), HtCA (I and J) and LOGACA (K and L) through high usage residues, labelled blue. Functional residues are annotated as follows: hotspot residues - h; interface residues - i; CO_2 binding pocket residues - c; Zn^{2+} coordinating residues - z. CmCA and LOGACA which have been previously characterized, are labelled in red.

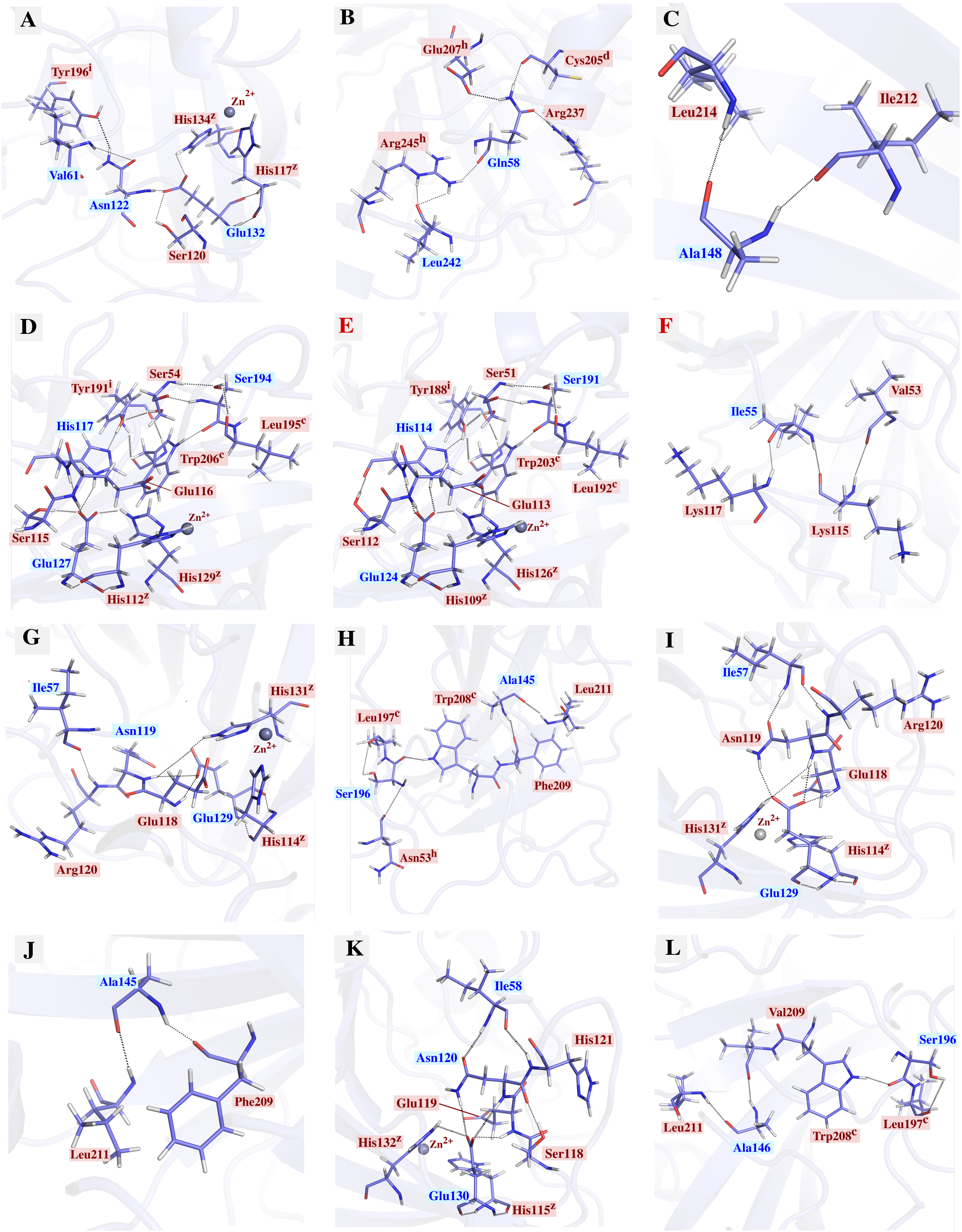

Figure S4: Intra-subunit hydrogen bond networks of NtCA (A-C), PhCA (D), PmCA (E and F), SlCA (G and H), SNbcCA (I and J) and SrCA (K and L) through high usage residues, labelled blue. Hotspot residues and CO_2 binding pocket residues are annotated h and c respectively. Functional residues are annotated as follows: hotspot residues - h; interface residues - i; intra-subunit disulfide Cys residues - d; CO_2 binding pocket residues - c; Zn^{2+} coordinating residues - z. PmCA which has been previously characterized, is labelled in red.

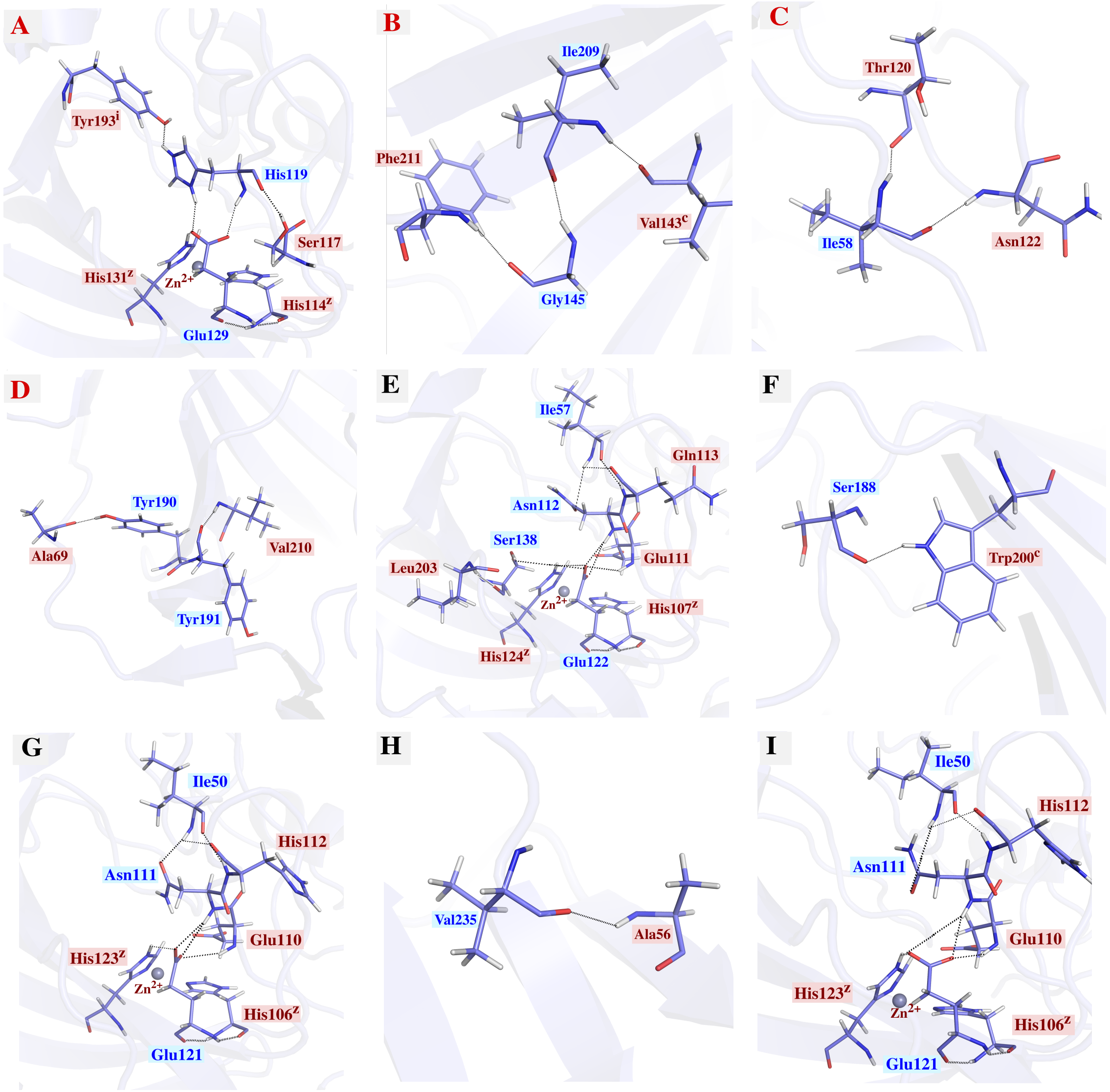

Figure S5: Intra-subunit hydrogen bond networks of TaCA (A-D), VaCA1 (E and F) and VaCA2 (G and H) and VdCA (I) through high usage residues, labelled blue. Hotspot residues and CO_2 binding pocket residues are annotated h and c respectively. Functional residues are annotated as follows: hotspot residues - h; interface residues - i; CO_2 binding pocket residues - c; Zn^{2+} coordinating residues - z. TaCA which has been previously characterized, is labelled in red.

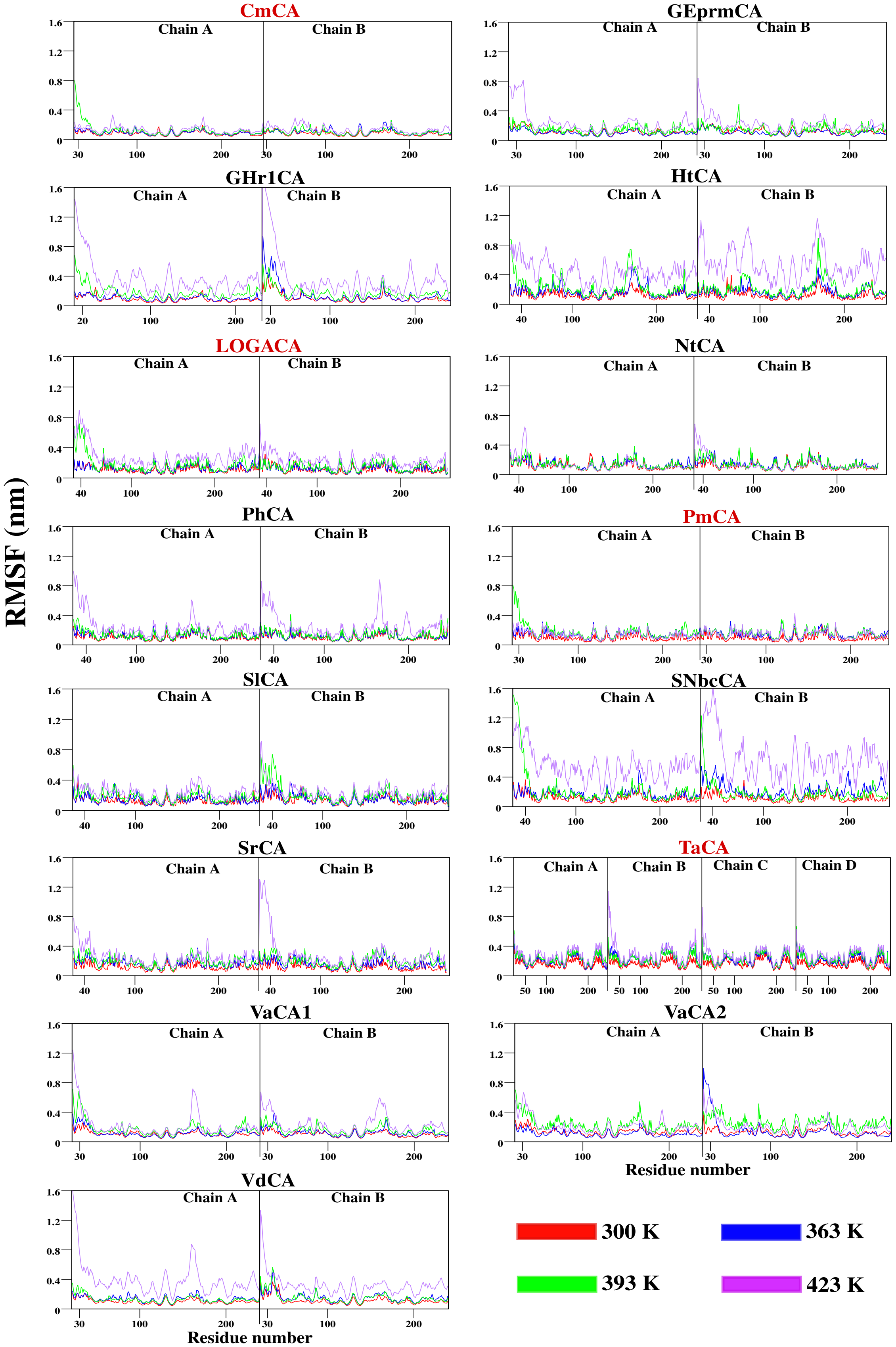

Figure S6: RMSF of protein structures at 300 K, 363 K, 393 K and 423 K. CmCA, LOGACA, PmCA and TaCA which have been previously characterized, are labelled in red.

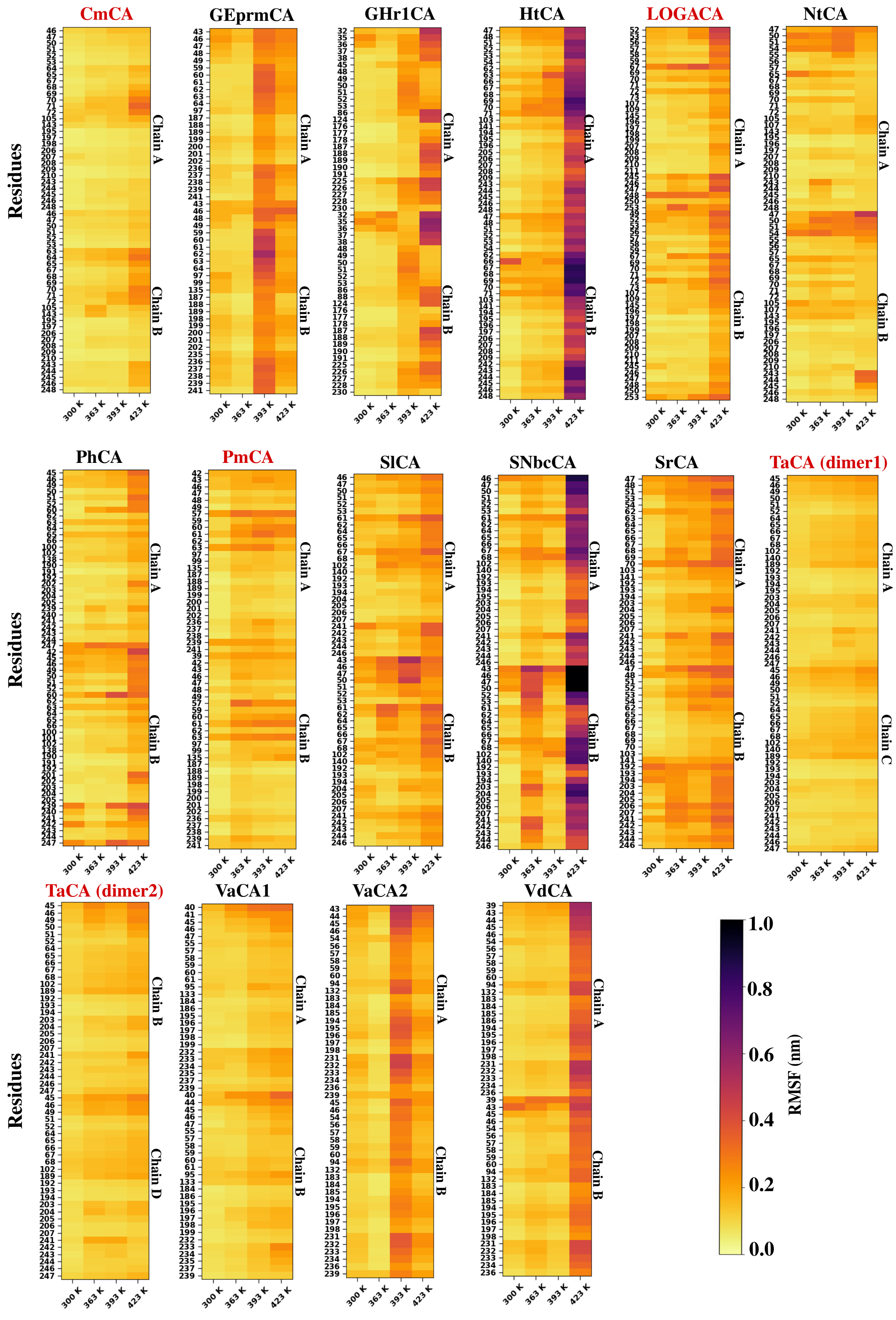

Figure S7: RMSF of interface residues of the α-CA proteins simulated at 300 K, 363 K, 393 K and 423 K. CmCA, LOGACA, PmCA and TaCA which have been previously characterized, are labelled in red.

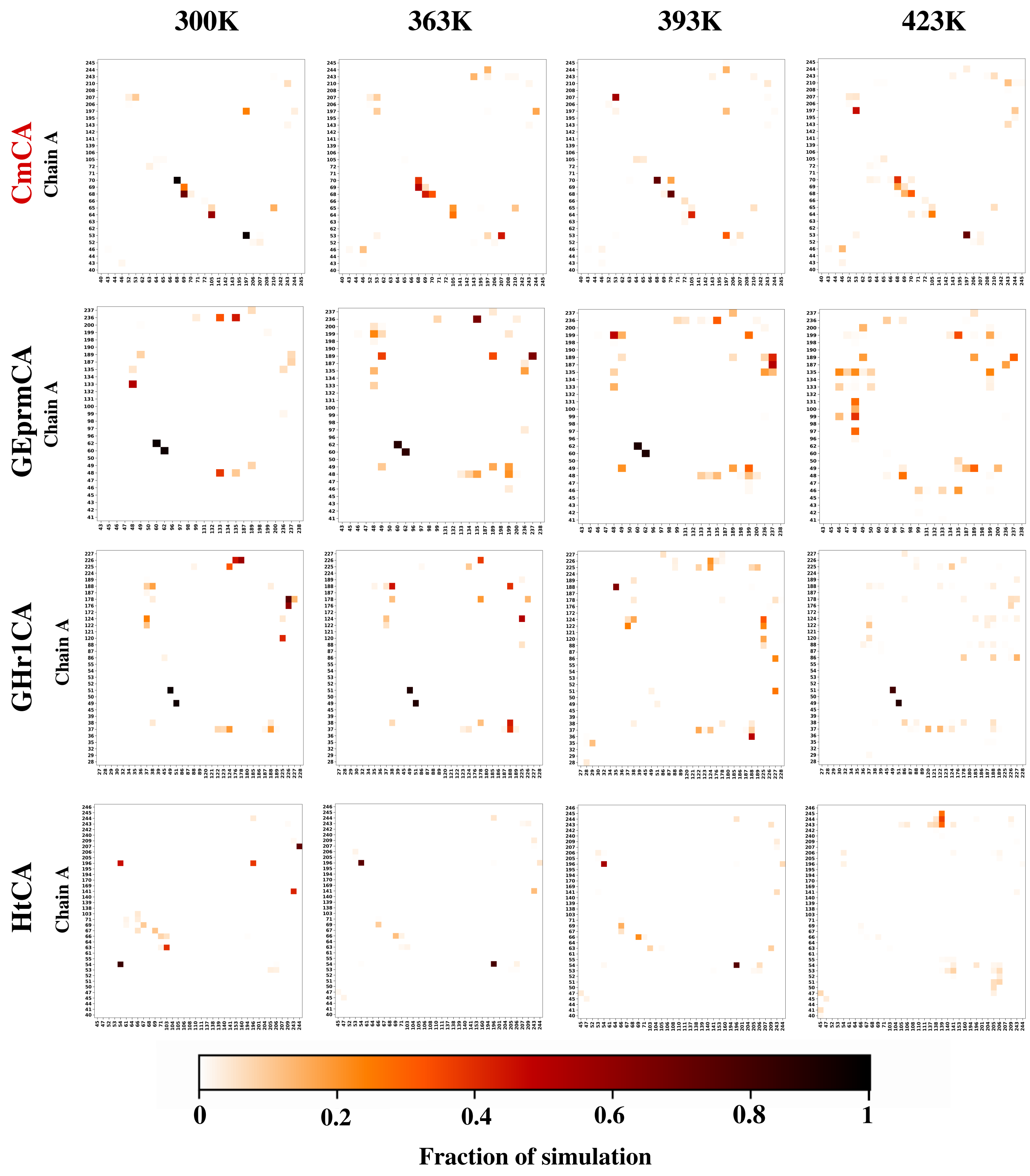

Figure S8: Inter-subunit hydrogen bonds networks of CmCA, GEprmCA, GHr1CA and HtCA at 300 K, 363 K, 393 K and 423 K. CmCA which has been previously characterized, is labelled in red.

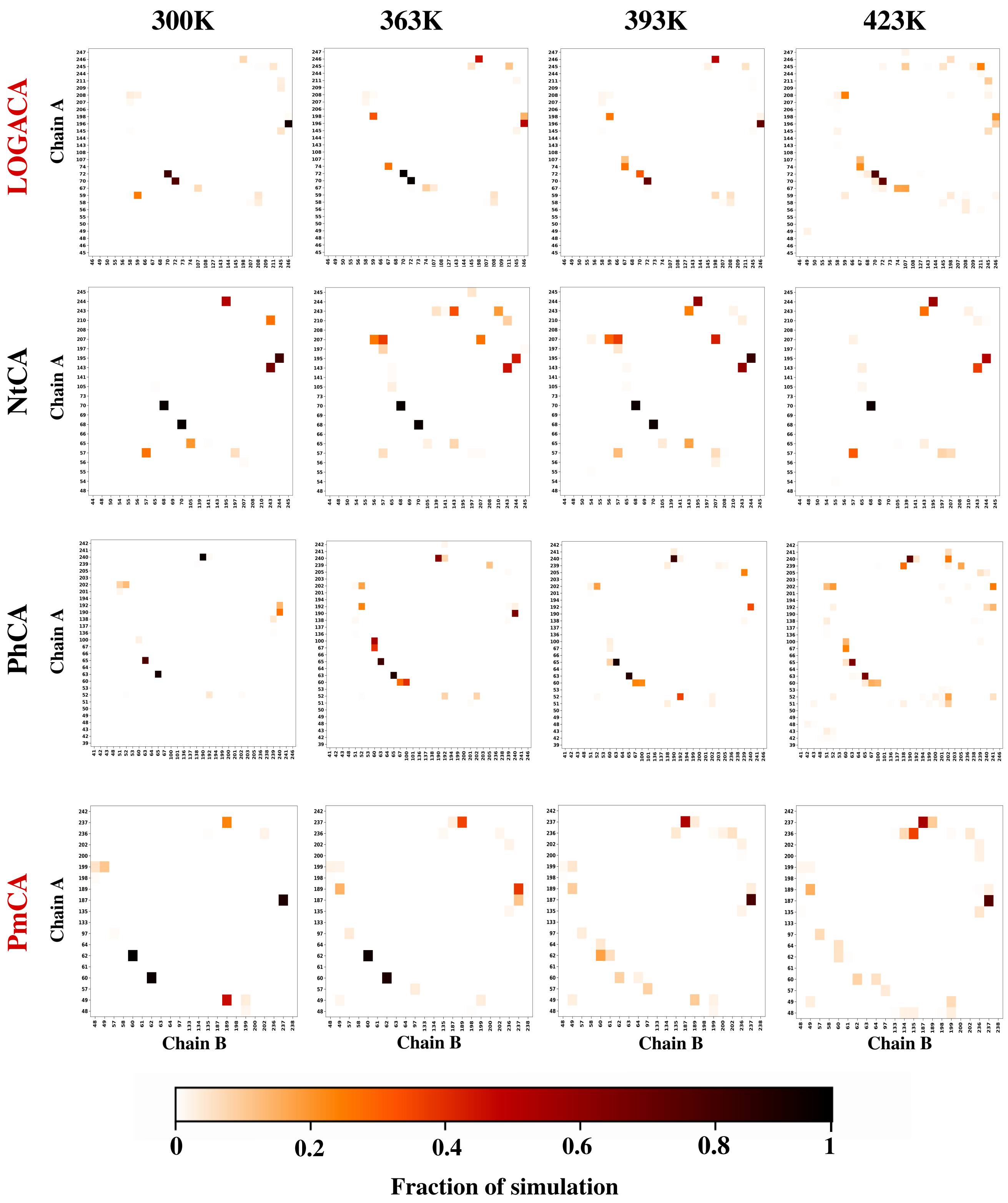

Figure S9: Inter-subunit hydrogen bonds of LOGACA, NtCA, PhCA and PmCA at 300 K, 363 K, 393 K and 423 K. LOGACA and PmCA which have been previously characterized, are labelled in red.

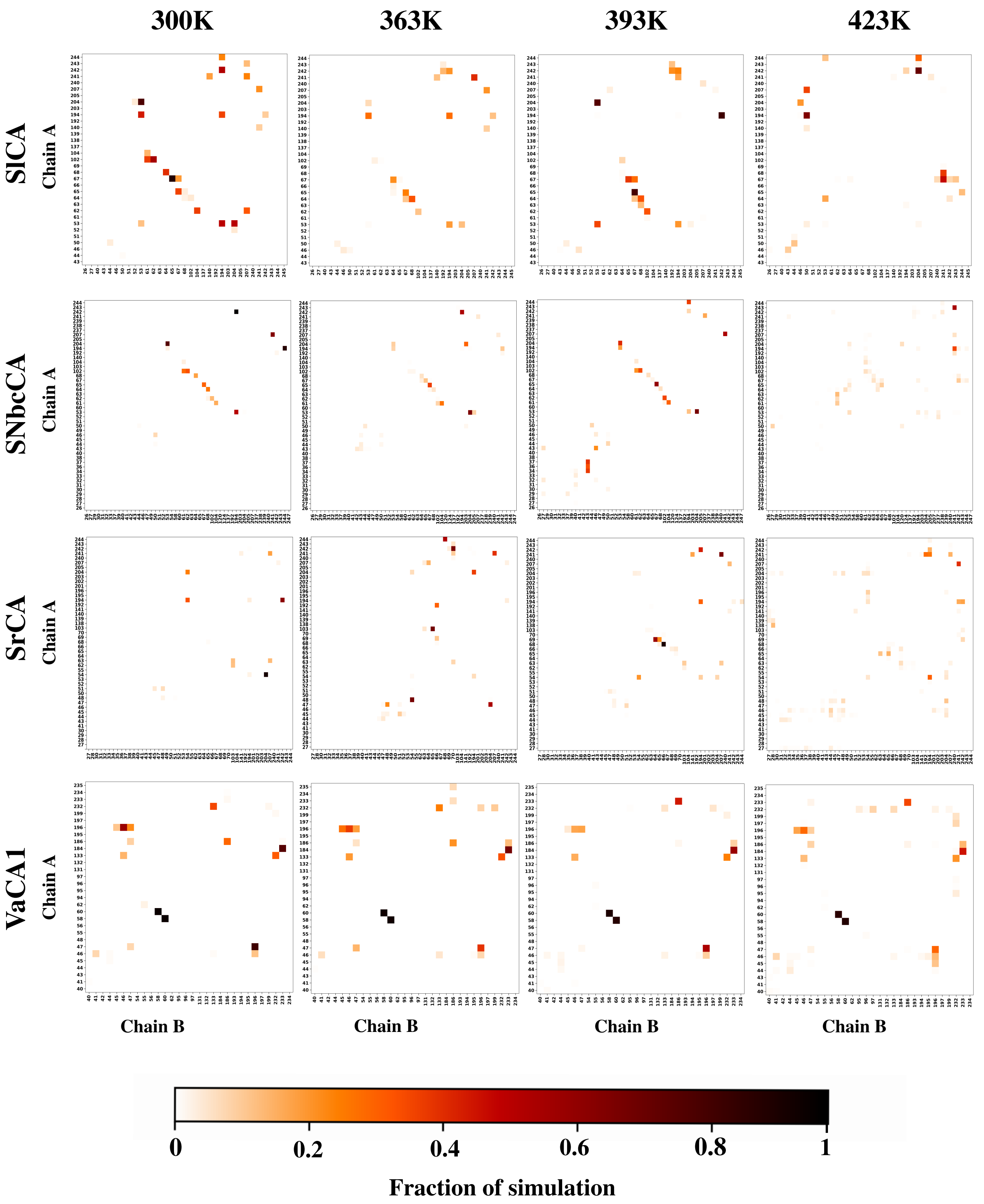

Figure S10: Inter-subunit hydrogen bonds of SlCA, SNbcCA, SrCA and VaCA1 at 300 K, 363 K, 393 K and 423 K.

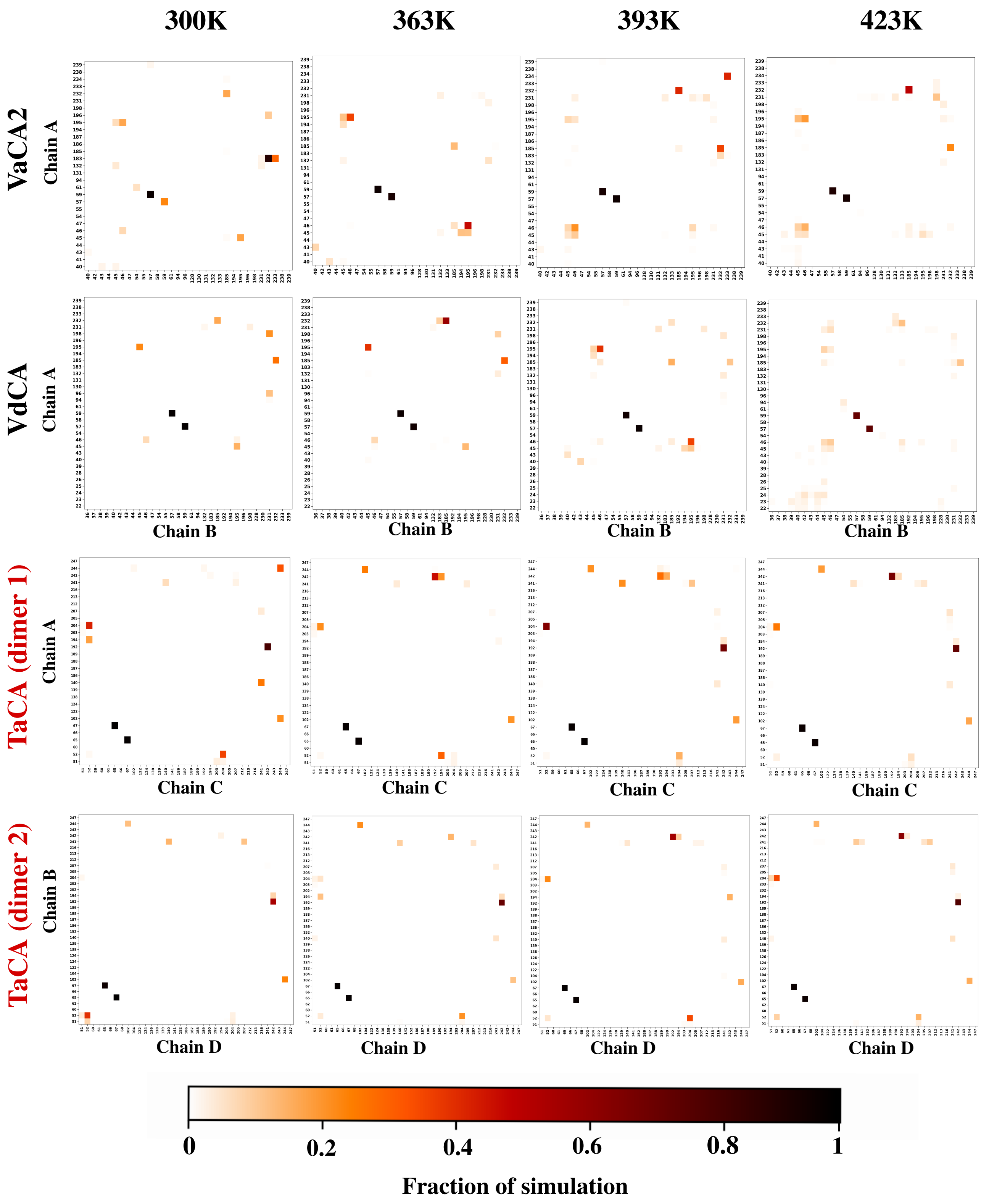

Figure S11: Inter-subunit hydrogen bonds of VaCA2, VdCA,and TaCA at 300 K, 363 K, 393 K and 423 K. TaCA which has been previously characterized, is labelled in red.
